# Measuring fidelity of delivery of the Community Occupational Therapy in Dementia-UK intervention

**DOI:** 10.1186/s12877-019-1385-7

**Published:** 2019-12-23

**Authors:** Holly Walton, Ildiko Tombor, Jane Burgess, Hilary Groarke, Tom Swinson, Jennifer Wenborn, Aimee Spector, Martin Orrell, Gail Mountain, Susan Michie

**Affiliations:** 1 Department of Applied Health Research, University College London, 1-19 Torrington Place, London, UK; 2 Department of Behavioural Science and Health, University College London, 1-19 Torrington Place, London, UK; 3 Research and Development Department, Goodmayes Hospital, North East London NHS Foundation Trust, Essex, UK; 4 Department of Clinical, Educational and Health Psychology, University College London, 1-19 Torrington Place, London, WC1E 7HB UK; 5 East Hertfordshire and Broxbourne Adult Disability Team, Hertfordshire County Council, Stevenage, UK; 6 Division of Psychiatry, Faculty of Brain Sciences, University College London, London, UK; 7 Institute of Mental Health, University of Nottingham, Nottingham, UK; 8 School of Health and Related Research, The University of Sheffield, Sheffield, UK

**Keywords:** Fidelity of delivery, Implementation, Dementia, Occupational therapy, Complex intervention

## Abstract

**Background:**

Interpreting data about intervention effectiveness requires an understanding of which intervention components were delivered and whether they were delivered as planned (fidelity of delivery). These studies aimed to develop a reliable measure for assessing fidelity of delivery of the Community Occupational Therapy in Dementia-UK intervention (COTiD-UK) (Study 1) and measure fidelity of delivery of COTiD-UK across sessions, sites and occupational therapists (Study 2).

**Methods:**

The studies used a longitudinal observational design nested within a multi-site randomised controlled trial. Where practicable, all intervention sessions were audio-recorded. Fidelity checklists and coding guidelines were developed, piloted and refined until good agreement was achieved between two coders. Ten percent of sessions were purposively sampled from 12 sites and 31 occupational therapists. Transcripts were coded using checklists developed in Study 1; 10% of sets of intervention session transcripts were double coded to ensure that agreement was maintained. Percentages of components that were delivered were calculated for each session, site and occupational therapist.

**Results:**

A reliable measure of fidelity of delivery for COTiD-UK was developed after several rounds of piloting and amendments. COTiD-UK was delivered with moderate fidelity across all six sessions (range: 52.4–75.5%). The mean range of fidelity varied across sites (26.7–91.2%) and occupational therapists (26.7–94.1%).

**Conclusions:**

A reliable, systematic method for measuring fidelity of delivery of COTiD-UK was developed and applied, and can be adapted for use in similar interventions. As COTiD-UK was delivered with moderate fidelity, there is a reasonable degree of confidence that intervention effects were attributable to COTiD-UK.

## Background

Dementia is a global health concern, with 115.4 million people expected to receive a diagnosis of dementia by 2050 [1]. Psychosocial interventions are developed to support people with dementia and their family carers in maintaining their quality of life. Interpreting the effectiveness of psychosocial interventions requires knowledge of the extent to which the intervention was delivered as planned (termed ‘fidelity of delivery’) [2–4]. Whilst psychosocial interventions for people with dementia may show promise, it is not always clear whether they have been delivered as planned [5].

One such intervention is the Community Occupational Therapy in Dementia (COTiD) intervention, which was first developed and delivered in the Netherlands [6, 7]. COTiD aimed to facilitate independence, meaningful activity and quality of life among people with mild to moderate dementia and their families [6]. COTiD was delivered to people with dementia and their family carers over 10 one hour sessions over five weeks [6]. The first four sessions focused on people with dementia and family carers choosing and prioritising meaningful activities that they wanted to improve, by interviewing both the person with dementia and family carer [6]. Adaptations to the home and environment were considered as part of these sessions. In the final six sessions, problem solving skills and coping strategies were developed to enhance the use of daily activities [6]. A single site randomised controlled trial (RCT) of COTiD found that both people with dementia and their family carers benefitted from the intervention [6, 7]. For example, people with dementia’s daily functioning and family carers’ sense of competence increased. Both groups experienced better quality of life than those who did not receive the intervention. However, when the intervention was replicated in Germany, no significant differences between intervention and control groups were found in a seven-site RCT [8, 9].

Differences in the effectiveness of COTiD could be attributed to: differing cultural contexts, translation of the intervention, differences in measures used, or differences in control groups. For example, in the German study, participants in the control group received a leaflet and one consultation visit from an occupational therapist [8], whereas participants in the control group in the Netherlands did not receive any occupational therapy [6]. It is also possible that differences could have been a result of how the intervention was delivered. Previous research has found that complex healthcare interventions are often not delivered as planned [10, 11]. In Germany, self-reported fidelity of delivery was reported to be 78% in the COTiD intervention group and 80% in the control group. A few intervention components were poorly delivered, suggesting that at least some aspects of COTiD had poor fidelity of delivery [8]. Previous studies of the COTiD intervention did not use the same measure of fidelity or ensure their reliability; therefore, fidelity of delivery cannot be accurately compared between Germany and the Netherlands.

To understand whether it is possible to deliver an intervention as planned and whether variable intervention effects could be the result of differences in intervention fidelity, it is important to measure fidelity. The current gold-standard is to record all sessions and select a representative sample to transcribe and compare against an intervention-specific checklist of intervention components [2, 3, 10, 12, 13]. To ensure that measurements are reliable, it is recommended that intervention transcripts are coded by multiple researchers [14]. To determine the trustworthiness of fidelity assessments, the psychometric quality of measures (e.g. reliability and validity) should be considered when developing and reporting them [15]. Fidelity should be monitored across providers, sites and participants to account for differences in context [10, 16].

The COTiD intervention was translated and adapted for the UK context [Wenborn et al., in preparation]. The Community Occupational Therapy in Dementia UK (COTiD-UK) intervention involves home and community-based occupational therapy with people with mild to moderate dementia and a family carer (referred to as ‘dyads’). COTiD-UK is designed to be delivered by occupational therapists. COTiD-UK was evaluated in a multi-centre, pragmatic, single blind RCT (COTiD-UK vs. treatment as usual) between October 2014 and July 2017. An embedded qualitative study involved qualitative interviews with occupational therapists and dyads to explore the experience of participating in the COTiD-UK intervention (for more information about COTiD-UK and the RCT, see [17]). Four hundred and sixty-eight dyads were recruited and randomly allocated to either the COTiD-UK intervention (*n* = 249) or a treatment as usual control group (*n* = 219).

Dyads in the intervention group received up to 10 h of COTiD-UK over approximately 10 weeks. Seven key sessions were delivered: 1. Introduction (occupational therapist introduced self and the COTiD-UK format), 2. Occupational Performance History Interview (OPHI) [18] (occupational therapist interviewed the person with dementia about their life, experiences and activities), 3. Ethnographic Interview (occupational therapist interviewed the family carer about their life and experience of providing care), 4. Summaries of interviews and observations (occupational therapist summarised the information gathered from the interviews, together with their own observations made within the context of an environmental and activity assessment), 5. Goal-setting (occupational therapist facilitated the dyad to create Specific, Measurable, Achievable, Realistic and Timed (SMART) goals), 6. Consultation and advice (occupational therapist enabled the carer to develop problem solving skills and provided other relevant advice and information), and 7. Evaluation (occupational therapist, person with dementia and family carer reviewed their progress in achieving the set goals). These six or seven sessions (depending on whether Summaries and Goal-setting were delivered separately or together) were collectively referred to as a ‘set’.

All occupational therapists received two days of face-to-face training in how to deliver the COTiD-UK intervention. They initially delivered the intervention to a ‘training dyad’, audio-recording all sessions as practicable. A COTiD-UK trainer listened and assessed the training dyad recordings to determine if the occupational therapist had achieved the necessary competencies to deliver COTiD-UK within the RCT. To enable occupational therapists to consolidate their COTiD-UK understanding, knowledge and skills and give opportunity for reflection, a follow-up training day was provided once they had delivered COTiD-UK. All occupational therapists participated in supervision throughout the delivery of COTiD-UK. Supervision models varied between peer, group and individual supervision, depending upon the availability of a COTiD-UK supervisor.

To understand the effectiveness of COTiD-UK, it was necessary to measure fidelity alongside the delivery of the trial. This research therefore aimed to: develop a measure for assessing fidelity of delivery of COTiD-UK (Study 1), and measure fidelity of delivery of COTiD-UK across intervention sessions, sites and occupational therapists (Study 2).

### Ethical approval (both studies)

All ethical and research governance requirements were followed. Ethical approval was obtained from: NRES Committee London – Camberwell St Giles, REC Reference number: 14/LO/0736. Encrypted audio-recorders were used and data transferred using encrypted memory sticks. Data were transcribed by a professional transcription company. All transcripts were fully anonymised and thus individuals were unidentifiable from the data or resulting outputs.

Study 1: The development of a measure to assess fidelity of delivery of COTiD-UK.

## Methods

### Design (both studies)

A longitudinal observational study nested within the COTiD-UK RCT was conducted across 12 of the 15 National Health Service (NHS) trust research sites that were involved in the trial, in England. COTiD-UK was delivered primarily by occupational therapists recruited from clinical services within participating organisations but in some cases was also delivered by research staff who were also occupational therapists. In these cases, occupational therapist researchers did not collect research data from their own participants.

### Procedure

#### Development of checklists

The development of fidelity of delivery checklists was informed by the methodology used to assess the fidelity of another psychosocial intervention for people living with dementia: Promoting Independence in Dementia (PRIDE) [19]. A team of behavioural scientists and occupational therapy research staff were involved in the development of the checklists.

The first step was to develop an intervention framework by identifying key components of the intervention. The COTiD-UK checklist template, the COTiD-UK training materials, and discussion with the COTiD-UK trainers informed this step. The COTiD-UK checklist was used by the occupational therapists to record the intervention provided in each session to each dyad as follows: 1) the date and length, 2) who was present, 3) location, 4) COTiD-UK components delivered, 5) travel time, 6) preparation time and 7) clinical recording time. Identifying key components involved going through each of the intervention materials and identifying active ingredients that occupational therapists needed to deliver in each session as part of the COTiD-UK intervention.

The resulting intervention framework outlined: 1) key targets, 2) key components (these were subsequently referred to as ‘appointment activities’ in the fidelity checklists), 3) the session(s) the component should be delivered in, and 4) whether the component is aimed at the person with dementia or the family carer or both (see Additional file 1).

The intervention framework was used to develop a fidelity checklist for each of the seven key intervention sessions. The ‘Summaries’ and ‘Goal-setting’ sessions were combined as they were often delivered together, resulting in six sessions for coding. The fidelity checklists included information on: 1) set identification number, 2) component number, 3) date of the session and 4) key components for each session. Key components were listed as ‘occupational therapist behaviours’ and components were recorded as ‘done’, ‘done to some extent’, ‘not done’ or ‘delivered in a different session’. To check for accuracy of content and comprehensibility of the components in the checklists, feedback on the intervention framework and checklists was then sought from the researchers and occupational therapy research staff involved in the fidelity assessments and training and delivery of COTiD-UK. To ensure that components accurately represented the intervention, components were discussed with the wider team which included occupational therapy research staff who were involved in the training of COTiD-UK providers. This feedback identified that key occupational therapy skills were missing from the framework (e.g. activity analysis, selection, adaptation and grading). To address this, we reviewed relevant occupational therapy literature [20] to identify and operationalise these skills for inclusion in the framework.

Coding guidelines for the fidelity assessment (see Additional file 2) were developed. The coding guidelines included: 1) instructions on how to code transcripts, 2) definitions for each component within each of the intervention sessions and 3) criteria for ‘done’, ‘done to some extent’ and ‘not done’ rating. Examples were also given to illustrate what should be coded as ‘done’, ‘done to some extent’, ‘not done’. Self-reported goal-setting forms (completed by the occupational therapists), the COTiD-UK leaflet and OPHI and Ethnographic interview questions were used to inform the coding guidelines and to facilitate decision making about what counted as ‘done’, ‘done to some extent’ and ‘not done’. For example: the topics listed within the OPHI and Ethnographic interview questions were used to distinguish between ‘done’ (open questions about 5+ topics), ‘done to some extent’ (open questions about 2–4 topics) or ‘not done’ (open questions about 0/1 topics).

#### Piloted and refined checklists and coding guidelines to improve reliability

Fidelity checklists and coding guidelines were then piloted and inter-rater agreement was calculated. Piloting initially involved two sets of sessions which were independently coded by three coding pairs. Three coders were naïve to the intervention and three coders had knowledge of it [21]. Disagreements and inter-rater agreement for each coding pair were calculated. If high levels of agreement were not achieved on the first set, the first author met with the coders to discuss disagreements and amend the coding guidelines accordingly before the second set of transcripts was coded. After all pairs completed the coding for these two sets, components that had poor reliability were identified and coding guidelines were amended.

After initial piloting, nine further sets were coded and the initial two sets were re-coded by one and/or two coding pairs (1st, 3rd, 4th, 5th authors). Discrepancies were discussed and coding guidelines and checklists were amended until good agreement was achieved.

The checklists and coding guidelines went through many amendments throughout various stages of the checklist development before being finalised. Changes were made to provide clarity and attempt to increase reliability. There were too many changes to provide a complete list, but some examples are given below. Some of the components relating to style of communication were difficult to code and amendments were made to operationalise these guidelines further. For example, the guidelines for ‘used open questions’ were changed from ‘asking open questions when appropriate’ to specifying how many topics would need to be discussed using open questions to count as ‘done’, ‘done to some extent’ and ‘not done’. Other components were difficult to code because of the way components were worded. Amendments were made to address these difficulties. For example, we merged three goal-setting components (setting goals for the person with dementia, family carer and both) to create two components that were more reliable to code (set at least one individual or joint goal for the person with dementia and family carer and developed these goals into SMART goals). Similarly, for other components we added extra information into the coding guidelines to reduce uncertainty and subjectivity for coders.

### Analysis

Inter-rater agreement was calculated using the Kappa statistic (κ) [22] and percentage agreement. To account for the ordinal nature of responses, weighted Kappa was used. This meant that ‘partial agreements’ were considered [23]. For example: some combinations of responses e.g. ‘done’ and ‘done to some extent’ are closer in agreement than others, e.g. ‘done’ and ‘not done’. A threshold of > 0.61 Kappa, established for three consecutive sets, was chosen after initial coding demonstrated that > 0.8 Kappa would be too difficult to achieve consistently. The > 0.61 Kappa threshold is considered ‘good’ agreement [24]. Kappa provides a conservative estimate of reliability [21]. Therefore, by lowering the threshold it was still possible to achieve good agreement whilst also accounting for chance agreement. Adjustments were made to the checklists and coding guidelines until good levels of inter-rater reliability were obtained.

## Results

### Development of checklists

**Fig. 1:**
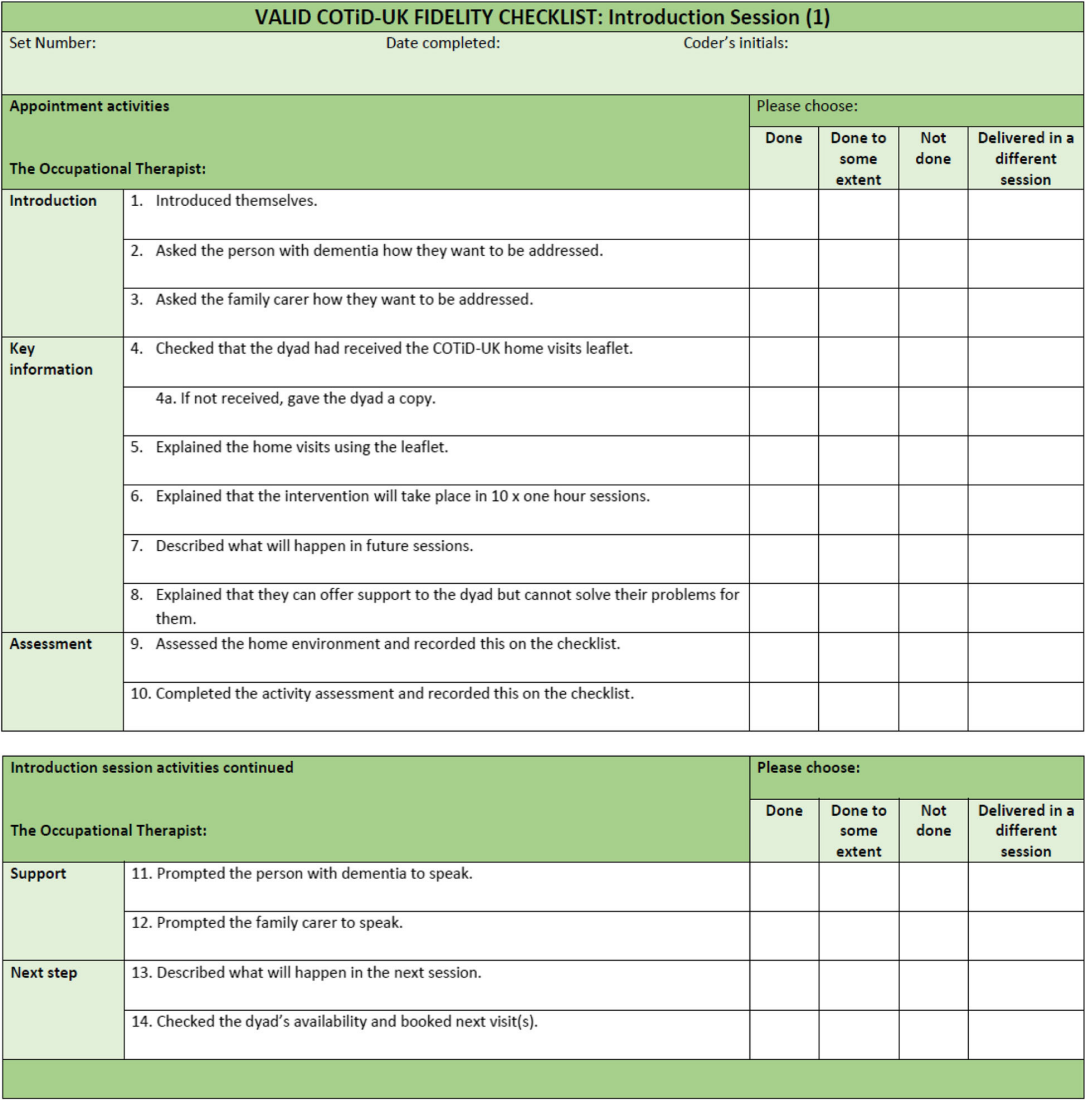
COTiD-UK Introduction (1) checklist

Six checklists (from seven sessions) were developed, each containing standardised intervention components (Introduction: 15 components, OPHI: 16 components, Ethnographic interview: 16 components, Summary and Goal-setting: 17 components, Consultation and advice: 15 components, Evaluation: eight components). See Fig. 1 for an example of the checklists. See Additional file 3 for all six checklists.

### Piloted and refined checklists and coding guidelines to improve reliability

Table 1 outlines the inter-rater agreement (weighted Kappa and percentage agreement) achieved for each session in the piloting phase. Good inter-rater agreement (κ > .61) was achieved after coding six Introduction transcripts (κ = 0.7), six OPHI transcripts (κ = 0.8–0.9), ten Ethnographic transcripts (κ = 0.7–0.8), 13 Summary and Goal-setting transcripts (κ = 0.8–0.9), eight Consultation and advice transcripts (κ = 0.6–0.9) and 11 Evaluation transcripts (κ = 0.7–0.8). For the Summary and Goal-setting session, >.61 Kappa was achieved three times in the last five sets (κ = 0.6–0.9). However, for this session good agreement (>.61 Kappa) was not achieved three times in a row due to an unequal distribution of responses, i.e. a large number of the same responses [25], which meant that weighted Kappa was moderate (κ = 0.4) but percentage agreement was very high (82.4%).

**Table 1:** Weighted Kappa and percentage agreement scores for each session by round of pilot coding, set of transcripts and coding pair

Linear weighted Kappa (percentage agreement)							
Set of transcripts	Coding pair	Introduction (1)	OPHI (2)	Ethnographic interview (3)	Summary and Goal-setting (4&5)	Consultation and advice (6)	Evaluation (7)
Initial piloting							
1	1a	0.79 (87)	0.66 (68.8)	0.48 (56.3)	0.5 (58)	6: 0.22 (35) 6 (2): 0.44 (65)	0.37 (44)
1	1b	0.93 (87)	0.40 (56.3)	0.2 (43.8)	0.23 (41.7)	6: 0.11 (58.8) 6 (2): 0.01 (41)	0.11 (33.3)
2	1b	0.65 (66.7)	0.27 (43.8)	0.57 (62.5)	0.28 (50)	6: 0.16 (47) 6 (2): 0.13 (35.3)	0.36 (55.6)
1	2	0.40 (60)	0.50 (62.5)	0.49 (56.3)	0.15 (37.5)	6: 0.5 (64.7) 6 (2): 0.08 (47)	0.16 (33.3)
2	2	0.50 (53.3)	0.68 (75)	0.81 (81.3)	0.42 (54)	6: 0.32 (41) 6 (2): 0.34 (47)	0.54 (66)
After initial piloting							
3	3	0.70 (80)	0.75 (81.3)	0.66 (75)	4: 0.78 (82.6) 5: 0.37 (60.9)	0.58 (82.4)	0.33 (55.6)
4	3	0.71 (73.3)	0.87 (87.5)	1.00 (100)	0.56 (59.6)	0.3 (58.8)	0.71 (87.5)
5	3	0.57 (66.7)	0.56 (68.8)	0.48 (62.5)	0.50 (69.6)	0.42 (56.3)	0.5 (50)
5	4	0.56 (60)	0.69 (81)	0.67 (75)	0.43 (65)	0.61 (68.8)	0.67 (75)
5	5	0.49 (66.7)	0.87 (87.5)	0.67 (75)	0.4 (56.5)	0.31 (62.5)	0.27 (37.5)
6	4	0.67 (73.3)^a^	0.87 (87.5)^a^	0.81 (87.5)	0.37 (52)	0.54 (81.3)	1.00 (100)
8^1^	4	0.7 (80)^a^	0.94 (93.8)^a^	0.55 (68.8)	0.46 (60.9)	0.63 (75)	0.73 (75)
12	4	0.66 (80)^a^	0.81 (87.5)^a^	0.87 (87.5)	0.77 (88.2)	0.93 (93.3)^a^	0.58 (75)
1	4	–	–	0.57 (70.6)	0.59 (70.6)	0.66 (73.3)^a^	1.00 (100)
2	4	–	–	0.81 (87.5)^a^	0.34 (58.8)	0.615 (80)^a^	0.39 (62.5)
9	4	–	–	0.66 (75)^a^	0.64 (76.5)^a^	–	0.74 (75)^a^
10	4	–	–	0.81 (81)^a^	0.76 (76.5)^a^	–	0.73 (75)^a^
11	4	–	–	–	0.49 (70.6)	–	0.77 (87.5)^a^
3	4	–	–	–	4: 0.94 (94.1)^a^	–	–
					5: 0.82 (88.2)^a^		
4	4	–	–	–	0.44 (82.4)^a2^	–	–

Note: Missing responses were accounted for in the analysis

^a^Indicates agreement > 0.61 was reached | 1Coding guidelines not changed after coding this set | 2Weighted kappa did not reach > 0.61 however > 70% agreement achieved five times in a row and > 0.6 kappa 3 times in last five sets Kappa low due to distribution of responses: lots of ‘done’ responses, despite only three disagreements

-: Agreement had already been reached and no further sessions needed to be coded until the 10% checks

Study 2: Measuring fidelity of delivery of COTiD-UK across sessions, sites and occupational therapists.

## Methods

### Sampling

**Fig. 2:**
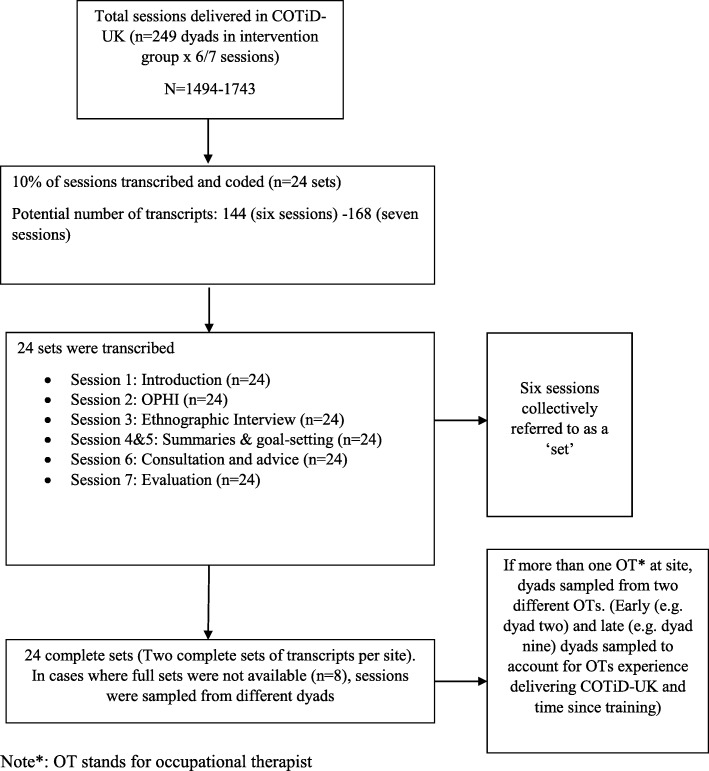
A flow chart to show the sampling strategy for this study, selected from the COTiD-UK trial

Figure 2 illustrates the sampling strategy. A sample of 10% of audio-recorded intervention sessions were purposively selected, transcribed and analysed, giving 24 sets. As each set included six or seven COTiD-UK sessions, depending on whether ‘Summary and Goal-setting’ sessions were delivered separately or together, the total number of sessions transcribed potentially ranged from 144 to 168. In cases where ‘Summary and Goal-setting’ were delivered separately, these are referred to in the results as Goal-setting 1 (Summary) and 2 (Goal-setting). These sets were purposively sampled from 12 of the 15 trial sites and 28 of the 31 occupational therapists. One of the 15 field work sites did not recruit any dyads, another was unable to provide the intervention and one did not have sufficient recordings for sampling; therefore they were omitted. Two sets were selected from each of the remaining 12 sites. Purposive sampling was used to ensure that we sampled from a range of occupational therapists from different sites, and to ensure that the transcripts selected were complete sets. To take occupational therapists’ prior clinical and specific COTiD-UK experience into account, recordings were sampled from different therapists and from dyads that were recruited early in the intervention period (e.g. an occupational therapist’s second dyad) and dyads that were recruited near the end of the intervention period (e.g. an occupational therapist’s ninth dyad). If no full sets were available (*n* = 8), sets which had the majority of sessions were sampled.

### Materials

The checklists developed in Study 1 were used to measure fidelity across sessions, sites and occupational therapists in Study 2. See Study 1 for more details.

### Procedure

Occupational therapists were asked to audio-record all intervention sessions provided that 1) consent for audio-recording was obtained and 2) it was practical to do so (e.g. sessions conducted in a home setting but not those involving community activities). Some sessions were not recorded due to some dyads not consenting to audio-recording at the recruitment stage (*n* = 8). Other reasons included: information technology difficulties (e.g. problems with using the recorder or downloading recordings) or dyads declining audio-recording on the day. Routine audio-recording was chosen to overcome the possibility that occupational therapists may self-select which sessions to record [14].

All data were coded for fidelity by the first author. Ten percent of sets were double coded by the fourth author to check that inter-rater agreement was maintained and to assess for coder drift. If inter-rater agreement was not maintained and coder drift occurred, disagreements were discussed to achieve consensus.

### Analysis

Descriptive statistics (mean %, range %) were calculated. These were compared across sessions, occupational therapists and sites.

Components were scored according to whether they were: ‘done’ (score 2), ‘done to some extent’ (score 1), ‘not done’ (score 0), ‘delivered in a different session’ (coded as ‘98’), ‘not done, not applicable’ (coded as ‘99’). A score of ‘done to some extent’ was coded when components were neither fully ‘done’, nor ‘not done’. For example, ‘done to some extent’ was coded if open questions were used to explore two to four topics rather than five or more topics for the OPHI/Ethnographic interviews. ‘Not done, not applicable’ was coded when the delivery of a component had previously been accounted for, or would be accounted for in the next session (if sessions were delivered together). For example, the component ‘assessed the home environment’ could be delivered in the first, second or third session. Therefore, when it was coded as ‘done’ in the first session, this was coded as ‘not done, not applicable’ in the second and third sessions. To provide a conservative estimate of fidelity and to ensure that fidelity was comparable across occupational therapists and sites, components that were not applicable or delivered in a different session were scored as ‘0’ (not done).

Components that required the absence of behaviour (e.g. ‘use of jargon’) were reverse coded. A total score and the percentage of the number of components delivered were calculated. A higher score and percentage indicated higher fidelity (80–100% high fidelity, 51–80% moderate fidelity and < 50% low fidelity) [3]. The percentages of sessions for which individual components were ‘done’, ‘done to some extent’, ‘not done’, ‘not applicable’ or ‘delivered in a different session’ were calculated.

## Results

### Quality of measures

Out of 2696 completed sessions (the total number of COTiD-UK sessions across all sites), 1409 audio-recordings were made (52.3%). Twenty-four sets of audio-recordings were chosen for transcription (*n* = between 144 and 168 transcripts, depending on whether six or seven sessions were delivered). From these transcripts, 137 transcripts were available and were coded, seven were missing and two were labelled incorrectly and could not be coded. Overall, 84 components were scored ‘not applicable’ (Introduction: 22, OPHI: 32, Ethnographic interview: 24, Summary and Goal-setting (1 and 2): 6 and 3, Consultation and advice: 2, Evaluation: 1), and 27 components were scored ‘not done, delivered in a different session’ (Introduction: 1, Ethnographic interview: 1, Summary and Goal-setting (1 and 2): 6 and 18, Consultation and advice: 1).

See Table 2 for weighted Kappa and percentage agreement across sessions. Inter-rater agreements for the 10% of sets that were double coded were all >.61, including the Introduction session (κ = 0.6–0.8), OPHI (κ = 0.8–0.9), Ethnographic interview (κ = 0.7–0.9), and Summary and Goal-setting (κ = 0.7–1). Agreement for the Consultation and advice session dropped below the required threshold for set eight (κ = 0.5), but then agreement was reached for sets 13 (κ = 0.8) and 16 (κ = 0.8). Agreement for the Evaluation session dropped below the required threshold for sets eight (κ = 0.5), 13 (κ = 0.3) and 14 (κ = 0.3), but then agreement was reached for sets 15 (κ = 1.0) and 16 (κ = 0.9). For set 24, agreement for the Consultation and advice (κ = − 0.1) and Evaluation sessions (κ = 0.2) were particularly low. In this set, the Consultation and advice session and Evaluation session were combined and delivered by the occupational therapist in one session and there was no clear distinction as to which components belonged to which session; therefore, it was difficult to code with high agreement.

**Table 2:** Weighted Kappa and percentage agreement for 10% of COTiD-UK data that were double coded during the main fidelity assessment

Set	Weighted Kappa (%)					
	Introduction (1)	OPHI (2)	Ethnographic interview (3)	Summary and Goal-setting (4&5)	Consultation and advice (6)	Evaluation (7)
8(^a^)	0.6 (66.7)	0.9 (93.8)	0.8 (81.3)	0.7 (82.4)	0.5 (73.3)	0.5 (75)
13	–	–	–	–	0.8 (86.7)	0.3 (62.5)
14	–	–	–	–	–	0.3 (75)
15	–	–	–	–	–	1 (100)
16 (^a^)	0.8 (86.7)	0.8 (81.3)	0.7 (81.3)	1 (100)	0.8 (80)	0.9 (87.5)
24 (^a^)	No transcript	0.8 (81.3)	0.9 (87.5)	0.7 (82.4)	−0.1 (33.3)	0.2 (37.5)

(^a^) Sets were selected for double coding

-: Agreement had already been reached and no further sessions needed to be coded until the next sampled session

No transcript – refers to sessions where transcripts were not available to code

### Measuring fidelity of delivery of the COTiD-UK intervention

Table 3 reports the fidelity of delivery scores across sessions, occupational therapists and sites.

**Table 3:** Fidelity of delivery scores (mean %, (range %)) for components delivered across COTiD-UK sessions across sites

Site	Session (Mean % (Range %))						
	Introduction (1)	OPHI (2)	Ethnographic interview (3)	Summary and Goal-setting (4&5)	Consultation and advice (6)	Evaluation (7)	Summary and Goal-setting (2) (4&5 (2))^b^
	52.4% (26.7–86.7) or 47.5^a^ (13.3–86.7)	75.5 (62.5–90.6)	71.9 (56.3–84.4)	71.7 (52.9–94.1)	65.6 (30–86.7)	69.0 (43.8–87.5)	44.1 (29.4–55.9)
A	26.7 (13.3^a^-40)	76.6 (75–78.1)	70.3 (62.5–78.1)	57.4 (52.9–61.8)	30.0 (30.0)	68.8 (56.3–81.3)	51.5 (47.1–55.9)
B	58.3 (53.3–63.3)	82.8 (78.1–87.5)	76.6 (75–78.1)	91.2 (88.2–94.1)	86.7 (86.7)	71.9 (68.8–75.0)	–
C	35.0 (33.3–36.7)	82.8 (75–90.6)	70.3 (56.3–84.4)	77.9 (73.5–82.4)	55.0 (53.3–56.7)	75.0 (75)	–
D	55.0 (53.3–56.7)	75.0 (68.8–81.3)	75.0 (68.8–81.3)	64.7 (61.8–67.7)	80.0 (80.0)	65.6 (56.3–75)	–
E	28.3 (26.7–30)	70.3 (62.5–78.1)	75.0 (68.8–81.3)	72.1 (52.9–91.2)	–	75.0 (75)	–
F	28.3 (13.3^a^-43.3)	71.9 (65.6–78.1)	67.2 (62.5–71.9)	77.9 (70.6–85.3)	63.3 (53.3–73.3)	78.1 (75–81.3)	–
G	68.3 (60–76.7)	73.4 (65.6–81.3)	68.8 (62.5–75.0)	83.8 (76.5–91.2)	71.7 (70–73.3)	65.6 (56.3–75)	–
H	68.3 (56.7–80)	78.1 (75–81.3)	68.8 (68.8)	73.5 (67.7–79.4)	71.7 (66.7–76.7)	65.6 (56.3–75)	–
I	26.7 (13.3^a^-40)	73.4 (71.9–75)	73.4 (65.6–81.3)	64.7 (64.7)	71.7 (70–73.3)	43.8 (43.8)	–
J	36.7 (33.3–40)	73.4 (68.8–78.1)	73.4 (65.6–81.3)	63.2 (55.9–70.6)	–	65.6 (62.5–68.8)	–
K	60.0 (53.3–66.7)	76.6 (68.8–84.4)	71.9 (71.9)	69.1 (58.8–79.4)	46.7 (46.7)	78.1 (75–81.3)	–
L	78.4 (70–86.7)	71.9 (65.6–78.1)	71.9 (68.8–75.0)	64.7 (64.7)	53.3 (53.3)	75.0 (62.5–87.5)	29.4 (29.4)

Note: Max fidelity (100%): Introduction: *n*=30, OPHI and Ethnographic interview: *n* = 32, Summary and Goal-setting: *n* = 34, Consultation and advice: *n* = 30, Evaluation: *n* = 16

Components that were N/A are coded as missing and thus scored ‘0’ in percentage calculations, therefore fidelity for individual sets may be underestimated

^a^Three session 1’s were missing – cannot tell fidelity of these. Can only tell if the observations have been carried out, thus leading to some sets having only 13.3% fidelity. Average without these data points also provided (without ^a^) | ^b^4&5(2) = second Summary and Goal-setting transcript when OTs have delivered 4&5 separately

-: No transcript available

To ensure site anonymity, site numbers have been shuffled up so that numbers 1–15 do not directly correspond to letters A-L

Overall, a mean of 52.4% (range: 30–86.7%) of components were delivered as planned in the Introduction session, 75.5% (range: 62.5–90.6%) in the OPHI session, 71.9% (range: 56.3–84.4%) in the Ethnographic interview, 71.7% (range: 52.9–94.1%) in the Summary and Goal-setting session, 65.6% (range: 30–86.7%) in the Consultation and advice session, and 69% (range: 43.8–87.5%) in the Evaluation session. The second Summary and Goal-setting session was delivered with low fidelity (44.1% of components; range: 29.4–55.9%). This shows that COTiD-UK was delivered with moderate fidelity overall.

The percentages of components that were delivered (‘done’, ‘done to some extent’, ‘not done’, ‘not applicable’, or ‘delivered in a different session’) in each session are reported in Additional file 4. Component numbers in Additional file 4 directly correspond with component numbers in the COTiD-UK checklists. For example, components that were frequently ‘not done’ for the Summary and Goal-setting session were: ‘checked they understood information from family carer’ (component five, *n* = 14, 58.3% not done), ‘summarised their own views’ (component six, *n* = 13, 54.2% not done) and ‘told participants to start to carry out activities’ (component 14, *n* = 13, 54.2% not done). Components that were frequently ‘done’ for the Summary and Goal-setting session were: ‘described what will happen in session’ (component one, *n* = 24, 100% done), ‘discussed potential activities’ (component seven, *n* = 24, 100% done), ‘set at least one goal’ (component eight, *n* = 22, 91.7% done) and ‘prompted the person with dementia and family carer to speak’ (component 15, *n* = 23, 95.8% done).

### Comparing fidelity of delivery across intervention sites and OTs

As Table 3 shows, fidelity of delivery for each session varied across sites (Introduction: 26.7–78.4%, OPHI: 70.3–82.8%, Ethnographic interview: 67.2–76.6%, Summary and Goal-setting: 57.4–91.2%, Consultation and advice: 30–86.7% and Evaluation: 43.8–78.1%) and occupational therapists (Introduction: 26.7–78.4%, OPHI: 62.5–90.6%, Ethnographic interview: 56.3–84.4%, Summary and Goal-setting: 52.9–94.1%, Consultation and advice: 30–86.7% and Evaluation: 43.8–81.3%). This shows that whilst COTiD-UK was delivered with moderate fidelity overall, not all occupational therapists and sites achieved moderate fidelity across all sessions.

## Discussion

### Key findings

In Study 1, a systematic method was used to create a reliable measure of fidelity and apply it to measure fidelity of delivery of the COTiD-UK intervention. Results found that the intervention was delivered moderately well across COTiD-UK sessions. This means that if the COTiD-UK trial finds that the intervention is associated with improved activities of daily living or quality of life, there can be a reasonable degree of confidence that these effects were the result of the planned intervention. If COTiD-UK is found not to be effective, these findings indicate that the intervention was either not effective, or influenced by other factors (e.g. lack of engagement or contamination within the usual care group). As fidelity of COTiD-UK was moderate, and not high, this indicates that some content was not delivered as planned, with variation noted across sites and occupational therapists. Findings may be used to explain the relationship between RCT outcomes and qualitative findings.

### Findings in relation to previous research

In addition to the fidelity of intervention delivery, the psychometric qualities of fidelity measures can inform the interpretation of intervention evaluation effects [15]. The findings from Study 1 are consistent with previous research indicating that it is difficult to achieve good inter-rater reliability when measuring fidelity [26, 27]. Many rounds of coding and amendments to the coding guidelines and checklists were required to reach good agreement. To help achieve agreement, clear definitions of components were developed and provided to make guidelines easier to use and limit subjectivity in responses, as recommended by previous research [10, 26, 28–30]. Furthermore, once agreement was maintained it was necessary to monitor fidelity throughout the fidelity assessment to identify coding drift. This importance of monitoring fidelity throughout the assessment is highlighted in Study 2, as there were instances where agreement dropped below the required threshold. Whilst this was a time-consuming process, monitoring of agreement throughout the fidelity assessment was found to be feasible.

The average fidelity of delivery of intervention components across the intervention sessions ranged from 52.4–75.5%. This shows that complex occupational therapy interventions for people with dementia, like other behavioural interventions, are not fully delivered as planned [10, 11, 14, 31].

Previous research indicates that fidelity often varies across sessions, sites and providers [10, 16]; the results of Study 2 are consistent with these findings. An explanation for differences in fidelity across sessions could lie in the degree of structure and/or provision of materials for some sessions. For example, occupational therapists were given example questions for the OPHI and Ethnographic interviews and were asked to fill in a goal-setting form for the Summary and Goal-setting session. The clear structure of these sessions and the provision of materials may have prompted the delivery of some components, thus increasing fidelity. Following delivery of the goal-setting components, the intervention was then tailored towards the dyads’ individual goals, which may have made it more challenging for occupational therapists to deliver the intervention with fidelity.

Differences across occupational therapists and sites could also reflect the difference in occupational therapists’ backgrounds and skills. All COTiD-UK providers were occupational therapists and will have shared experience of occupational therapy training. Yet, individual occupational therapists differ in the level of professional experience in working with people with dementia and their family carers, which could have also contributed towards differences in fidelity. Additionally, differences in fidelity may be attributable to different work environments, since COTiD-UK took place across multiple sites. For example, some occupational therapists may have had peers (whom they could seek advice and support from) whereas others may have delivered the intervention with limited peer support. All occupational therapists within COTiD-UK were offered supervision. However, supervision models differed between peer, group and individual supervision, which could have influenced the fidelity of delivery and contributed towards the observed differences.

Differences across occupational therapists and sites could also reflect the levels of tailoring towards individual needs in different sessions. Occupational therapy is a complex dynamic process that comprises multiple practices, the implementation of which is individualised, with the relationship between the person(s) and therapist being fundamental as the process necessitates the active involvement of the person(s) and therapist working in partnership [32]. Hence, occupational therapists may have decided that some components were not relevant in certain situations with particular dyads. Alternatively, occupational therapists may have needed to adapt to a situation that arose on the day (e.g. managing a crisis), which may have influenced fidelity. Although occupational therapists were asked to include explanatory notes within the COTiD-UK checklists, the extent to which this was completed varied, and so limited the degree to which this data could be used to inform the fidelity analysis.

This fidelity assessment identified that some aspects of COTiD-UK were predominantly delivered as standalone sessions to all participants, whereas other aspects were delivered more flexibly. Triangulation of findings with the COTiD-UK checklists and supervision records indicated that there were many reasons for this, depending on the session or aspect of the intervention. For example, the OPHI, Ethnographic Interview and Summary and Goal-setting sessions were delivered as planned to all participants. On the other hand, components of the Introduction and Evaluation sessions were sometimes integrated at the beginning or end of other sessions. Also, in practice, the occupational therapist often delivered some of the Introduction components either: by telephone when setting up the appointment; or prior to turning the audio-recorder on; with the latter situation usually influenced by needing to establish rapport and confirm consent. Not all participants received a Consultation and advice session, and in fact ‘Consultation and advice’ may better be described as a technique rather than a stand-alone session. In practice, Consultation and advice was often integrated within other sessions; or may not have been delivered at all if it did not relate to the goals set. These findings could indicate that the ‘OPHI’, ‘Ethnographic Interview’, and ‘Summary and Goal-setting’ sessions are the core aspects of COTiD-UK. Conversely, the ‘Introduction’, and ‘Evaluation’ components may be delivered more flexibly - which hinders recording them to assess fidelity, and techniques such as ‘Consultation and advice’ are often integrated to other sessions, or not delivered at all, depending on the individual dyads’ situation and needs.

The results from Study 2 found that fidelity for COTiD-UK was lower than COTiD in Germany; the former was based on audio-recorded data, and the latter was based on provider self-report [8]. The use of different methods may explain differences in fidelity, as self-report may lead to biases such as social desirability or difficulties remembering what was delivered [33]. The checklists used to measure fidelity in this study were different than those used in Germany: in COTiD-UK, more specific components were assessed, operationalised as specific activities that occupational therapists could deliver. For example, in this study, OPHI was a key session which contained 16 components (e.g. open questions, use of visual objects). In Germany, ‘interviewing the person with OPHI’ was included as one component [9]. Without using the same reliable measures across intervention contexts, it is difficult to compare whether interventions were delivered as planned and whether differences in delivery explain possible differences in intervention effects.

Alternatively, a further explanation for differences in delivery across providers and sites, but also any differences in effectiveness between different COTiD trials may be attributable to the design of the intervention(s). Researchers have proposed key differences between pragmatic trials (closer to real life situations) and explanatory trials (highly controlled situations) [34, 35]. The Pragmatic-explanatory continuum indicator summary (PRECIS) tool proposes ten aspects on which pragmatic and explanatory trials may differ, including: participant eligibility criteria, flexibility in the intervention and comparison conditions, practitioner expertise for the intervention and comparison conditions, follow up intensity, primary trial outcomes, participant compliance with prescribed intervention, practitioner adherence to study protocol and analysis of primary outcome [35]. COTiD-UK has not been rated against the PRECIS criteria, but would likely score highly on many of these domains, including practitioner adherence to study protocol (as fidelity was closely monitored throughout the COTiD-UK trial), primary trial outcomes, follow-up intensity and analysis of primary outcomes. Other aspects of COTiD-UK may be nearer to the middle of the pragmatic-explanatory continuum [35]. For example: flexibility in intervention conditions (as the intervention was specified but allowed for occupational therapists tailoring the intervention towards individuals’ goals and needs) and practitioner expertise (as COTiD-UK was delivered by a number of occupational therapists with differing levels of experience). This may explain variations in the delivery of COTiD-UK across providers and participants. This may also contribute towards understanding of differences between COTiD-UK and previous COTiD trials; if they were implemented differently.

### Limitations

A limitation of this research is that fidelity may have been underestimated in some instances, since some aspects of COTiD-UK were not captured using audio-recording. These included: components of COTiD-UK delivered outside of the home, telephone calls, interactions which occurred after the session had finished and non-verbal interactions.

Furthermore, ‘not applicable’ components were scored as ‘not done’ in the analysis. This decision would particularly affect those sessions where ‘not applicable’ responses were high, including the Introduction Session (*n* = 22), OPHI (*n* = 32) and Ethnographic interview (*n* = 24). There were a large number of ‘not applicable’ responses for the Introduction session as many Introduction sessions were delivered at the same time as the OPHI; thus rendering components about the next visit ‘not applicable’. Furthermore, there were a large number of ‘not applicable’ responses for the interviews, as ‘assessing the home environment’ was included on all three checklists. Therefore, it is likely that the fidelity of these sessions, in particular, may be underestimated.

Whilst data on the treatment as usual pathways were collected for the control group in COTiD-UK, fidelity of COTiD-UK specific components was not measured in the control condition. Therefore, it is not known exactly what the participants in the control groups received. This undermines the ability to draw conclusions from intervention effects [3]. This is particularly important given the differences in control groups in the RCT of COTiD in the Netherlands and Germany, with one control group receiving one occupational therapy visit and the other not receiving any [6, 8]. If control groups vary on the type of support delivered, this could influence the effectiveness of the intervention.

One limitation is that we have only measured fidelity in this study. Other factors could also affect intervention outcomes. For example, one unknown aspect is the extent to which dyads engaged with the intervention (e.g. their understanding and ability to perform skills or strategies learnt during the intervention, and whether they can put their plans into practice in daily life) [15]; thus, differential engagement cannot be ruled out as a possible factor which may influence COTiD-UK effects [3]. Similarly, given that this trial had many ‘explanatory’ characteristics, it is possible that implementation in a real-world setting may differ to that of a trial setting. This may be one reason why we observed many ‘not applicable’ responses in our fidelity assessment.

### Implications

The fidelity checklists developed for this study are specific to this intervention and do not necessarily apply directly to other interventions. However, the method was adapted from measures developed to measure the fidelity of an intervention to improve independence in dementia (PRIDE [19]. As such, the transferability of the method across these studies suggests that it could potentially be applied to develop reliable measures of fidelity for other complex, psychosocial interventions for people with dementia, or interventions more generally.

The study results can also be used to identify problematic components (those which were frequently ‘done to some extent’ or ‘not done’) within each session. For example, further training for the Summary and Goal-setting session may need to focus on: ‘checking that they understood the information provided by the family carer’ (component five), ‘summarising own views from observations and assessments’ (component six), ‘adapting the activity’ (component 10), ‘providing information about environmental barriers’ (component 11), or ‘telling participants that they could start to carry out activities to meet the goals’ (component 14). Booster training sessions may be needed to enhance fidelity for difficult to deliver components or instances where fidelity is particularly low.

For researchers and policymakers, findings can inform decisions about whether and how COTiD-UK should be implemented on a wider scale.

### Future research

The checklists produced by this study could be useful for investigating the extent to which COTiD-UK is delivered with fidelity across varying settings and cultures.

Although fidelity was compared across occupational therapists, differences in fidelity were not compared across their experience, gender and age. Further research could investigate these differences.

Larger fidelity studies will enable multilevel modelling to statistically test the differences in fidelity of delivery across providers and sites and their association with intervention effectiveness. This would help to determine which components of COTiD-UK may be most effective and therefore important to deliver.

## Conclusion

A systematic method for measuring fidelity has been developed and used to reliably assess fidelity of delivery of COTiD-UK. COTiD-UK was delivered with moderate fidelity overall, however, its delivery varied across occupational therapists and sites. Findings from this study inform the interpretation of effects reported in the COTiD-UK RCT and qualitative research. Overall, there can be a reasonable degree of confidence that any intervention effects can be attributed to the COTiD-UK intervention. If COTiD-UK is found to not be effective, these findings indicate that COTiD-UK was either not effective or that other factors which were not measured in this study may have influenced effectiveness (e.g. lack of engagement or contamination from usual care). If COTiD-UK is found to be effective, these findings indicate that the intervention content has the potential to support people with dementia and their family carers to maintain independence, engage in meaningful activity and enhance quality of life. Using these findings to understand the delivery of COTiD-UK across sites and occupational therapists may facilitate the interpretation of both RCT and qualitative findings and provide potential explanations for the level of effectiveness of COTiD-UK.

## Supplementary information

**Additional file 1.** COTiD-UK framework

**Additional file 2.** Final coding guidelines

**Additional file 3.** COTiD-UK checklists

**Additional file 4.** Percentage of transcripts in which individual components were delivered fully, to some extent, or not at all per COTiD-UK sessions

## Data Availability

The datasets generated and/or analysed during the current study are not publicly available due to participant confidentiality but are available from the corresponding author on reasonable request.

## Abbreviations

COTiD: Community Occupational Therapy in Dementia
COTiD-UK: Community Occupational Therapy in Dementia- United Kingdom
NHS: National Health Service
OPHI: Occupational Performance History Interview
PRIDE: Promoting Independence in Dementia
RCT: Randomised Controlled Trial
SMART: Specific, Measurable, Achievable, Realistic, Timed
UK: United Kingdom
