# Supplementary material for: Measuring fidelity of delivery of the Community Occupational Therapy in Dementia-UK intervention
Source: BMC Geriatr. 2019 Dec 23;19:364. doi: 10.1186/s12877-019-1385-7 (PMC6929510; doi:10.1186/s12877-019-1385-7)
Supplement: Supplementary file 1 — Additional file 1. COTiD-UK framework [file 12877_2019_1385_MOESM1_ESM.docx]

**Additional files**

**Additional file 1– COTiD-UK framework**

COTiD-UK is an occupation based intervention that aims to promote independence, meaningful activity and quality of life for people with dementia and their family.

OT = occupational therapist

| **Framework** | **Key targets** | **Key components*** | **Skill / session** | **Target audience** | | N/A |
| --- | --- | --- | --- | --- | --- | --- |
|  |  |  |  | PwD | FC (FS) |  |
| Key information | Introductions | OT introduces themselves | Introduction | √ | √ |  |
|  |  | OT asks person with dementia how they want to be addressed | Introduction | √ | √ |  |
|  |  | OT asks family carer how they want to be addressed | Introduction | √ | √ |  |
|  | Explain what intervention involves | OT checks that the dyad has received the COTiD-UK home visits leaflet | Introduction | √ | √ |  |
|  |  | OT gives dyad a copy if not received   - May be not applicable | Introduction | √ | √ |  |
|  |  | OT explains the home visits using the leaflet if applicable | Introduction | √ | √ |  |
|  |  | OT explains that the intervention will take place over 10 hours in 10 x one hour sessions, | Introduction | √ | √ |  |
|  | Explain aim of sessions | Explain aim of current session | Summaries and goal-setting, Consultation and advice, Evaluation | √ | √ |  |
|  |  | Explain aim of future/next sessions | OPHI, Ethnographic interview, Summaries and goal setting, Consultation and advice,  Evaluation | OPHI  √ | Ethno  graphic  √ |  |
|  | Explain role boundaries | OT explains that they can support the partnership between them and family carer but cannot solve | Introduction, Summaries and goal-setting, Consultation and advice, Evaluation |  | √ |  |
|  | Provision of information | OT provides information about the condition | Consultation and advice, Evaluation |  | √ |  |
|  |  | OT provides information about behavioural features | Consultation and advice, Evaluation |  | √ |  |
|  |  | OT provides information about potential support available | Consultation and advice, Evaluation |  | √ |  |
| Assessment and tailoring | Environment assessment | OT assesses home environment (Checklist) (NB cannot measure with recordings)   - May be not applicable | Introduction and/or OPHI,  Ethnographic interview | √ |  |  |
|  | Activity assessment | OT completes Activity assessment (Checklist) (NB cannot measure with recordings)   - May be not applicable | Introduction | √ | √ |  |
| Analysis | Activity analysis | OT identifies the skills required to achieve the activity | Summaries and goal-setting | √ | √ |  |
|  | Environmental analysis | OT provides information about the causes of problems | Summaries and goal-setting | √ | √ |  |
|  |  | OT gives explanations for behaviour | Summaries and goal-setting | √ | √ |  |
|  |  | OT gives ideas for modifications | Summaries and goal-setting | √ | √ |  |
| Communication techniques | Questions | OT uses open questions when appropriate | OPHI, Ethnographic interview | √ | √ |  |
|  |  | OT prompts the person to provide more details about their responses (can you explain a bit more, can you give me an example?) | OPHI, Ethnographic interview, Consultation and advice | √ | √ |  |
|  |  | OT doesn’t ask any questions that raises the person’s anxiety (reverse coded in checklist – distress) | OPHI, Ethnographic interview | √ | √ |  |
|  | Language | OT uses jargon/technical language (repeating words used, making use of metaphors) | OPHI, Ethnographic interview, Consultation and advice, Evaluation | √ | √ |  |
|  | Search for meaning | OT asks about the feeling and meaning of situations and activities | OPHI, Ethnographic interview | √ | √ |  |
|  | Summarise and seek confirmation | OT summarises the information provided by the person | OPHI, Ethnographic interview, Summaries and goal-setting | √ | √ |  |
|  |  | OT checked they understood the information provided by the person | OPHI, Ethnographic interview, Summaries and goal-setting | √ | √ |  |
|  | Balance in conversation | The family carer is able to express his/her reactions and feelings | Consultation and advice |  | √ |  |
|  |  |  |  |  |  |  |
|  | Interpretation | OT interprets the person’s story (*?) | OPHI, Ethnographic interview, Summaries and goal-setting | √ | √ |  |
|  | Reflect on own experiences | OT does not tell their own story (reverse coded – spoke about themselves) | OPHI, Ethnographic interview | √ | √ |  |
|  | Offer solutions | OT does not offer any solutions (reverse coded – told the person what to do) | OPHI, Ethnographic interview | √ | √ |  |
|  | Use objects | OT uses objects if appropriate | OPHI, Ethnographic interview | √ | √ |  |
| Intervention activities. | Support | OT gives the person with dementia the opportunity to speak (ask questions/give views) | Introduction; all | √ | √ |  |
|  |  | OT gives the family carer the opportunity to speak (ask questions/give views) | Introduction; all | √ | √ |  |
|  | Feedback | OT summarises the person with dementia’s story (from OPHI) | Summaries and goal-setting | √ | √ |  |
|  |  | OT summarises the family carer’s story (What came out of the ethnographic interview) | Summaries and goal-setting | √ | √ |  |
|  |  | OT summarises the occupational therapist’s story (from activity and environment observations) | Summaries and goal-setting | √ | √ |  |
|  | Goal setting | A SMART (specific, measureable, achievable, realistic, time) goal is created with the person with dementia | Summaries and goal-setting | √ |  |  |
|  |  | A SMART (specific, measureable, achievable, realistic, time) goal is created with the carer | Summaries and goal-setting |  | √ |  |
|  |  | A joint SMART (specific, measureable, achievable, realistic, time) goal is created with the dyad | Summaries and goal-setting | √ | √ |  |
|  |  | OT summarises the final list of goals agreed | Summaries and goal-setting | √ | √ |  |
|  |  | OT explains that participants should start working on the goal | Summaries and goal-setting | √ | √ |  |
|  |  | OT supports the family carer to formulate a goal using the How can you achieve that? formula | Consultation and advice |  | √ |  |
|  | Using activity | OT uses prepared cards with some important activities and possible goals identified through the OPHI and ethnographic interviews (Analysing potential activities) | Summaries and goal-setting | √ | √ |  |
|  |  | OT uses blank cards to include new activities and goals that arise during the discussion (Analysing potential activities) | Summaries and goal- setting | √ | √ |  |
|  |  | OT selects the best activity to engage the client (selecting appropriate activities) | Summaries and goal- setting | √ | √ |  |
|  |  | OT produces a new activity to achieve the outcome (synthesising new activities) | Summaries and goal- setting | √ | √ |  |
|  |  | OT changes the demands of an activity (e.g. changes tools equipment, material) (adapting chosen activities) | Summaries and goal- setting | √ | √ |  |
|  |  | OT grades the activity (adapting chosen activities)- manipulates factors so that the activity becomes more difficult or easy – social, emotional, cognitive, perceptual or physical demands) | Summaries and goal- setting | √ | √ |  |
|  |  | OT sequences the activity (adapting chosen activities) – The OT designs a sequence of different but related activities (to increase or decrease demand on participants) to suit the participants needs. | Summaries and goal- setting | √ | √ |  |
|  | Environmental adaptation | OT recommends changes to the clients’ environments (physical, cultural, institutional, and social) to influence motivation and help performance. | Summaries and goal-setting | √ | √ |  |
|  | Operationalising goals | OT supports the family carer to think about potential actions | Consultation and advice |  | √ |  |
|  |  | OT supports the family carer to select an action | Consultation and advice |  | √ |  |
|  | Problem analysis | OT creates a problem analysis together with the family carer | Consultation and advice |  | √ |  |
|  | Summarise | OT summarises the activities | Consultation and advice |  | √ |  |
|  | Review goals | OT reviews behaviour goal(s) using COTiD-UK goal setting form | Evaluation | √ | √ |  |
|  |  | OT uses the COTiD-UK goal setting form to record the goals | Evaluation | √ | √ |  |
|  | Planning ahead. | OT helps the dyad to identify goals for addressing future needs | Evaluation | √ | √ |  |
|  |  | OT supports the dyad in how to continue making progress without the OT | Evaluation | √ | √ |  |
| Admin | Future plans | OT checks dyad availability and books next visit(s) | Introduction/all sessions | √ | √ |  |
|  |  | OT explained what will happen next | Evaluation | √ | √ |  |
|  |  | OT reminds dyad of 12 week follow up and not to tell researcher that they received the COTiD-UK intervention | Evaluation | √ | √ |  |
