# Supplementary material for: Measuring fidelity of delivery of the Community Occupational Therapy in Dementia-UK intervention
Source: BMC Geriatr. 2019 Dec 23;19:364. doi: 10.1186/s12877-019-1385-7 (PMC6929510; doi:10.1186/s12877-019-1385-7)
Supplement: Supplementary file 2 — Additional file 2. Final coding guidelines [file 12877_2019_1385_MOESM2_ESM.docx]

**Additional file 2 - Final coding guidelines**

**COTiD-UK Intervention: Fidelity checklist coding guidelines**

**About the checklists**

The checklists represent the 7 key skill sessions delivered in the VALID Intervention:

1. Introduction
2. OPHI Interview
3. Ethnographic interview

4 & 5) Summaries and Goal setting (2 sessions, combined, as many occupational therapists deliver these in the same session)

1. Consultancy and advice
2. Evaluation

These checklists detail what appointment activities the occupational therapists should have delivered in each session.

Each checklist has a series of appointment activities. Some activities happen in every session, whereas others are unique to one session only.

**Abbreviations**

OT= Occupational therapist

OPHI interview = Occupational performance history interview

COTiD-UK = Community occupational therapy in dementia UK

SMART goal = Specific, measurable, achievable, realistic and timed goal

**Transcription codes**

T = Occupational therapist

P = Participant

C = Family carer

**How do I fill out the checklists?**

Please:

- Read through the coding guidelines
- Read through the table guidelines for the session that you are working on to familiarise yourself with the session outline
- Read the transcript once all the way through to familiarise yourself with it
- Read the transcript again
- Record the set number, date that you are completing the checklist and your initials on the top of the checklist
- Go through the checklist appointment activities one by one
- Use track changes and comments function to add a code (intervention activity) to the transcript (word document) to demonstrate evidence for each appointment activity.
- The same evidence can be used to support more than one appointment activity.
- Please also add comments about the strength of the evidence (this will be helpful when deciding to what extent it has been delivered and for discussions between coders).
- For each skill and for every item on the checklist, please tick whether it was:
  - **Done**
  - **Done to some extent**
  - **Not done**

**Delivered in a different session**

- After coding all skill sessions, please go back through the components that were **‘not done’** and check if these have been delivered in any other session. If so, please tick ‘delivered in a different session’ and write the number of the skill session that it was delivered in.
  - Note: This is only applicable if a component is specific to one session only. If skills are more general and should be delivered in more than one skill session (e.g. explaining the aim/communication skills) this should not be coded.
  - Please do not code if ‘done to some extent’ in a different session

**More than one skill delivered in one session**

- If more than one skill has been delivered in one session, please split the transcript into the two skills and code the corresponding checklists accordingly. For example: if Introduction and OPHI skills are combined, please code the introduction checklist up to the beginning of the OPHI session.
- In these cases, if components apply to both sessions (e.g. setting the date of the next session) please code done for both.

**How do I decide which score to give?**

- Please choose **‘done’** if all aspects of the activity have clearly been delivered
- Please choose **‘done to some extent’** if the activity has been partially delivered, or if you think that the OT needed to do more.

For example:

*“Explained that they can offer support but cannot solve problems*”, if the OT has explained that they are there to offer support, but have not told the dyad that they cannot solve the problems for them then this would be ‘done to some extent’.

*“Helped the person with dementia to create a SMART goal”.* If the OT helped the person to create a goal, but it only met some of the SMART criteria, e.g. that it was specific and measurable, but not achievable, realistic or timed, choose ‘done to some extent’

- Please choose **‘not done’** if there is no evidence throughout the transcript that this has been delivered to the dyad or if this was not necessary.

For example:

*“Prompted the family carer to identify solutions for these problems”.*  If there is no evidence throughout the transcript that the family carer was helped to identify solutions for problems which they identified, choose ‘not done’.

- If you think that any component is **not applicable**, please choose ‘not done’ and write ‘N/A’ in the box.

**What are the criteria for scoring intervention components?**

Please see the below tables (one per session) for a list of appointment activities, along with their definitions, scoring instructions, and rationale for choosing ‘done’, ‘done to some extent’ and ‘not done’ for each session.

Please ensure that you are looking at the correct session coding guidelines.

**Saving the coded transcripts and checklists**

Please save the files with _ [your initials] and date at the end. For example: Setx checklists _HW 01092016 and Set x – Skill x_ HW 01092016

Please return the saved transcripts and checklists to me before we meet to discuss discrepancies.

| **Coding guidelines for Session 1: Introduction** | | | | |
| --- | --- | --- | --- | --- |
| **Framework component** | **Appointment activity** | **Definition (if needed)** | **Scores** | **Rationale for scores** |
| **Introduction** | 1. Introduced themselves.   Note: OT may have already have introduced their name before recording. | Introduction should include their name, professional role (occupational therapist) and role as part of the VALID research programme | **Done** | OT gave their name, professional role and role as part of the VALID research programme |
|  |  |  | **Done to some extent** | OT gave 1 or 2 of the three introduction criteria (name, professional role or role as part of VALID programme) |
|  |  |  | **Not done** | OT did not give their name, professional role or role as part of the VALID programme |
|  | 1. Asked the person with dementia how they want to be addressed. | (Appointment activity self-explanatory) | **Done** | OT asked the person with dementia how they would like to be addressed |
|  |  |  | **Not done** | OT did not ask the person with dementia how they would like to be addressed |
|  | 1. Asked the family carer how they want to be addressed. | (Appointment activity self-explanatory) | **Done** | OT asked the family carer how they would like to be addressed |
|  |  |  | **Not done** | OT did not ask the family carer how they would like to be addressed |
| **Key information** | 1. Checked that the dyad had received the COTiD-UK home visits leaflet. | (Appointment activity self-explanatory) | **Done** | OT checked whether the leaflet has been received |
|  |  |  | **Not done** | OT did not check |
|  | 4a. If not received, gave the dyad a copy. | Conditional on previous activity – provided copy of leaflet if do not have.  **Note:** If already received, score not done and write not applicable. | **Done** | OT gave dyad a copy |
|  |  |  | **Not done** | OT did not give dyad a copy,  **OR**  Dyad already had a copy and it was not necessary to give one (can also make a note that Not applicable) |
|  | 1. Explained the home visits using the leaflet.   Note: Cannot tell if using leaflet, therefore code content, **using leaflet** | Explaining the home visits includes giving information about:   - What is occupational therapy? - What do occupational therapists do? - What are the home visits? - What happens next? (Note: Assume that this happens before the session – assume done) | **Done** | OT gave information about all four topics. |
|  |  |  | **Done to some extent** | OT gave information about 2 or 3 of the topics. |
|  |  |  | **Not done** | OT gave information about 0 or 1 of the topics. |
|  | 1. Explained that the intervention will take place in 10 x one hour sessions. | Explained the number and duration of sessions (e.g. 10 one hour sessions) | **Done** | OT explained the number and duration ( 10 one hour sessions) |
|  |  |  | **Done to some extent** | OT explained the number of sessions  **Or**  OT explained the duration of sessions |
|  |  |  | **Not done** | OT did not explain the number or duration of sessions |
|  | 1. Described what will happen in future sessions. | Future sessions include: Interview with person with dementia (OPHI interview), interview with family carer (ethnographic interview), summaries and goal-setting, consultation and advice and evaluation)   - **Interview with person with dementia (OPHI):** This involves a chat with the person with dementia to gain information about their life story. - **Interview with family carer (Ethnographic)**: This session involves a chat with the family carer to gain information about their life story - **Summaries and goal setting:** This session involves the OT summarising the information gathered in the OPHI and ethnographic interviews and also their own observations from assessments and then using this information to help the dyad set goals to work on throughout the programme - **Consultation and advice**: This session involves a chat with the family carer to work out what is working well and what is not working and to support the family carer to think about how to achieve those goals. - **Evaluation:** This session consists of reviewing the behavioural goals and helping the dyad to identify ways to continue making progress after the programme.   (For example: The OT talks about seeing them both individually to find out about their stories, observing a task, observing the environment, coming back together to draw on goals and work on those that have been developed). | **Done** | OT described what will happen in all future sessions |
|  |  |  | **Done to some extent** | OT described what will happen in some future sessions |
|  |  |  | **Not done** | OT did not describe what will happen in any future sessions |
|  | 1. Explained that they can offer support to the dyad but cannot solve their problems for them. | Explained that they are able to offer support (or help the dyad) but cannot solve the dyad’s problems for them.  Note: If the OT makes it clear in the session that they are supporting/helping the dyad and not telling them what to do, can code | **Done** | OT explained that they can support the partnership between the dyad but cannot solve problems |
|  |  |  | **Done to some extent** | OT explained that they can support but did not explain they cannot solve problems  **Or**  OT explained they cannot solve problems but did not explain that they can support the partnership, |
|  |  |  | **Not done** | OT did not explain they can support but cannot solve problems |
| **Assessment** | 1. Assessed the home environment and recorded this on the checklist.   Note: Cannot tell if recording on checklists therefore code content | Assessing the home environment is when the OT looks around the home environment and assesses it. Talking/Asking about the environment is not sufficient. But, if there is evidence that the OT goes to do a home assessment this can be coded (for example: ‘I will just have a look around your home’) **Measured using OT checklist (JB will tell us if this has been done)** | **Done** | OT assessed the home environment and recorded this on the COTiD-UK checklist |
|  |  |  | **Done to some extent** | OT assessed some but not all of the home environment  **Or**  OT assessed home environment but did not record it on the checklist |
|  |  |  | **Not done** | OT did not conduct a home assessment |
|  | 1. Completed the activity assessment and recorded this on the checklist.   Note: Cannot tell if recording on checklists therefore code content | An activity assessment is when the OT observes and assesses the person doing an activity) with the person. **Measured using OT checklist** | **Done** | OT completed an activity assessment and recorded this on the COTiD-UK checklist |
|  |  |  | **Done to some extent** | OT completed the activity assessment but did not record it on the checklist |
|  |  |  | **Not done** | OT did not complete an activity assessment |
| **Support** | 1. Prompted the person with dementia to speak | Prompted the person with dementia to speak, for example: ask questions or give views.  **Note:** If person with dementia speaks a lot without prompting, choose ‘not done and write N/A’  Note: This is different from ‘prompting for more information’. This component refers more to whether the participant has asked the participant if they have any questions (e.g. at the end of the session), or for more information on their views on decisions  E.g. ‘Do you have any questions?’/’What’s your views on that?’  Note: If one example of stopping speaking (to move the session on), can still code ‘done’, code ‘done to some extent’ if multiple examples of stopping speaking. | **Done** | OT prompted person with dementia to speak on **most appropriate occasions** |
|  |  |  | **Done to some extent** | OT prompted person with dementia to speak on some but not all appropriate occasions |
|  |  |  | **Not done** | OT did not prompt person with dementia to speak |
|  | 1. Prompted the family carer to speak | Prompted the family carer to speak, for example: ask questions or give views.  **Note:** If family carer speaks a lot without prompting, choose ‘not done and write N/A’  Note: This is different from ‘prompting for more information’. This component refers more to whether the participant has asked the participant if they have any questions (e.g. at the end of the session), or for more information on their views on decisions  E.g. ‘Do you have any questions?’/’What’s your views on that?’  Note: If one example of stopping speaking (to move the session on), can still code ‘done’, code ‘done to some extent’ if multiple examples of stopping speaking. | **Done** | OT prompted family carer to speak on **most appropriate occasions** |
|  |  |  | **Done to some extent** | OT prompted family carer to speak on some but not all appropriate occasions |
|  |  |  | **Not done** | OT did not prompt family carer to speak |
| **Next step** | 1. Described what will happen in the next session. | The OT described what will happen in the next session (OPHI interview, or home environment observation or activity observation)   - **OPHI interview**: This involves a chat with the person with dementia to gain information about their life story.   If skills 1 and 2 are delivered in the same session, choose not done (N/A) and code for skill 2 only | **Done** | OT described what will happen in the next session |
|  |  |  | **Done to some extent** | OT gave a session label but does not describe what will happen in the next session |
|  |  |  | **Not done** | OT did not describe what will happen in the next session |
|  | 1. Checked the dyad’s availability and booked next visit(s).   Note: If any indication that this has happened, can code. | The OT checked when the dyad would be available for the next visit and arranged the next visit.  If skills 1 and 2 are delivered in the same session, choose not done (N/A) and code for skill 2 only | **Done** | OT checked when the dyad would be available for the next visit and arranged the next visit |
|  |  |  | **Done to some extent** | OT checked when the dyad would be available but did not arrange the next visit  **Or**  OT arranged the next visit but did not check the dyad were available |
|  |  |  | **Not done** | OT did not check when the dyad would be available or arrange the next visit |
|  |  |  |  |  |

| **Coding guidelines for Session 2: OPHI interview** | | | | |
| --- | --- | --- | --- | --- |
| **Framework component** | **Appointment activity** | **Definition** | **Scores** | **Rationale for scores** |
| **Key information** | 1. Described what will happen in the current session. | Described what will happen in the current session (OPHI interview, or home observation or activity observation, or ethnographic interview)   - **OPHI interview:** This involves a chat with the person with dementia to gain information about their life story. | **Done** | OT described what will happen in the current session |
|  |  |  | **Done to some extent** | OT gave a session label but does not describe what will happen in the current session |
|  |  |  | **Not done** | OT did not describe what will happen in the current session |
| **Assessment** | 1. Assessed the home environment and recorded this on the checklist.   Note: Cannot tell if recording on checklists therefore code content | Assessing the home environment is when the OT looks around the home environment and assesses it. Talking/Asking about the environment is not sufficient. But, if there is evidence that the OT goes to do a home assessment this can be coded (for example: ‘I will just have a look around your home’). **Measured using OT checklist (JB will tell us if this has been done)**  **If you have coded ‘done’ for this component in first session, choose not done (N/A)** | **Done** | OT assessed the home environment and recorded this on the COTiD-UK checklist |
|  |  |  | **Done to some extent** | OT assessed some but not all of the home environment.  **Or**  OT assessed home environment but did not record it on the checklist |
|  |  |  | **Not done** | OT did not conduct a home assessment |
| **Communication techniques** | 1. Used open ended questions.   *Note: Use OPHI cards for scoring this | OT used open questions to ask about the person with dementia’s:   - Daily routine - Role - Environment - Leisure - Activity/occupational choices - Critical life events   Note: questions must be open questions  An open question is a question that cannot have a ‘yes/no answer’. For example. ‘Did you enjoy x?’ would be a closed question, but ‘How did you find doing x?’ would be an open question. | **Done** | OT used open questions to ask about at least 5 topics |
|  |  |  | **Done to some extent** | OT used open questions to ask about 2-4 topics |
|  |  |  | **Not done** | OT used open questions to ask about none or one topic. |
|  | 1. Prompted the person with dementia to provide more details about their responses. | When participants have provided an answer to a question, the OT has asked a further question to prompt the participant to go into more details about their answer. For example: ‘Can you explain a bit more?’ ‘Could you give me an example?’ Note these prompts can be subtle, for example: if the OT asks questions to prompt more information from a certain topic. | **Done** | OT asked for more details when appropriate most of the time |
|  |  |  | **Done to some extent** | OT asked for more details **sometimes but not always**, |
|  |  |  | **Not done** | OT **did not ask for** more details |
|  | 1. Asked questions which caused distress (*) | Questions which cause distress refer to those topics or issues that may cause undue distress to either the person, family carer or OT. Could include the continued questioning of sensitive topics, for example bereavement. | **Done** | OT asked questions which caused distress and continued questioning the person with dementia or family carer about sensitive topics. |
|  |  |  | **Not done** | OT did not ask distressing questions |
|  | 1. Used jargon or technical language (*) | Jargon or technical language is unexplained terminology which may not be understandable to members of the general public. This includes OT terminology. For example: ‘graded’/’environmental assessment’, ‘SMART goal’, ‘OPHI’, ‘problem analysis’ ‘action planning’, ‘activity synthesis’ or condition specific terms/terminology | **Done** | OT **used** jargon or technical language 4 or more times |
|  |  |  | **Done to some extent** | OT **used** jargon or technical language 2-3 times |
|  |  |  | **Not done** | OT **did not use** jargon or technical language. |
|  | 1. Asked the person with dementia to explain what the meaning of situations and activities were. | OT asks the person to explain what it means to them to do a certain activity or be in a certain situation (e.g. can you tell me what doing x means to you?’).  Example: The OT asks ‘How does it make you feel when you do some gardening?’  Can be either general or specific activities | **Done** | OT asked questions on the meaning of most activities or situations |
|  |  |  | **Done to some extent** | OT asked questions on the meaning of some activities or situations |
|  |  |  | **Not done** | OT did not ask questions on the meaning of activities or situations |
|  | 1. Summarised the information provided by the person with dementia. | Throughout the session, the OT summarised the information provided by the person with dementia by putting the information into their own words  Example: ‘So you have told me that you garden two times a week and that you enjoy it’ | **Done** | OT summarised the information provided by the person with dementia 4 or more times |
|  |  |  | **Done to some extent** | OT summarised the information provided by the person with dementia 1-3 times |
|  |  |  | **Not done** | OT did not summarise the information provided by the person with dementia |
|  | 1. Interpreted the information provided by the person with dementia (*) | The OT interpreted (made an inference or assumption) the information based on what they think that the person with dementia is saying e.g. ‘what I think you mean is…’  Example: (After the person says they like gardening), the OT says: ‘I’m guessing that you are an outdoor person’ | **Done** | OT interpreted the information provided by the person with dementia more than 4 times |
|  |  |  | **Done to some extent** | OT interpreted the information provided by the person with dementia 3 or 4 times |
|  |  |  | **Not done** | OT did not interpret the information or interpreted the information less than two times |
|  | 1. Checked that they understood the information provided by the person with dementia. | The OT asks the person with dementia if they understood the information correctly e.g. ‘Is that right?’ ‘Did I understand that correctly?’  Example: (After the person says they like gardening), the OT says: ‘Am I right in thinking that you enjoy gardening?’ | **Done** | OT checked that they understood the information provided by the person with dementia 4 or more times |
|  |  |  | **Done to some extent** | OT checked they understood the information provided by the person with dementia 2-3 times |
|  |  |  | **Not done** | OT did not check they understood the information provided by the person with dementia or checked the information provided by the person with dementia once |
|  | 1. Spoke about themselves. (*) | The OT told the person with dementia about their own life. (e.g. ‘I always quite enjoyed doing x, until…’). This must be more than just a natural response to a remark, e.g. (P: I like chocolate, OT: Me too!). | **Done** | OT spoke about themselves in detail more than 4 times |
|  |  |  | **Done to some extent** | OT spoke about themselves in detail 3 to 4 times |
|  |  |  | **Not done** | OT did not speak about themselves or spoke about themselves less than two times |
|  | 1. Told the person with dementia what to do. (*) | The OT told the participants what to do rather than helping them to decide what to do for themselves e.g. ‘you should do this…’ | **Done** | OT told the person with dementia what to do |
|  |  |  | **Not done** | OT did not tell the person what to do |
|  | 1. Used visual objects found in the person with dementia’s home to gather information (E.g. photos, garden, sewing, cakes, and paintings). | Visual objects could include photos, gardens, sewing, cakes, and paintings.  The OT prompted/led the use of the person with dementia’s objects or used objects to gather information if the opportunity comes up (e.g. participant shows object to OT). | **Done** | OT prompted the use of objects in conversation to gather information |
|  |  |  | **Done to some extent** | OT initiates but does not facilitate the use of objects  Or  OT facilitates but does not initiate the use of objects |
|  |  |  | **Not done** | OT did not use objects |
| **Support** | 1. Prompted the person with dementia to speak. | Prompted the person with dementia to speak, for example: ask questions or give views.  Note: If person with dementia speaks a lot without prompting, choose ‘not done and write N/A’  Note: This is different from ‘prompting for more information’. This component refers more to whether the participant has asked the participant if they have any questions (e.g. at the end of the session), or for more information on their views on decisions  E.g. ‘Do you have any questions?’/’What’s your views on that?’  Note: If one example of stopping speaking (to move the session on), can still code ‘done’, code ‘done to some extent’ if multiple examples of stopping speaking. | **Done** | OT prompted person with dementia to speak on **most appropriate occasions** |
|  |  |  | **Done to some extent** | OT prompted person with dementia to speak on some but not all appropriate occasions |
|  |  |  | **Not done** | OT did not prompt person with dementia to speak |
| **Next steps** | 1. Described what will happen in the next session. | Described what will happen in the next session (E.g. Ethnographic interview, activity observation, environment observation)   - **Ethnographic interview:** This session involves a chat with the family carer to gain information about their life story | **Done** | OT described what will happen in the next session |
|  |  |  | **Done to some extent** | OT gave a session label but does not describe what will happen in the next session |
|  |  |  | **Not done** | OT did not describe what will happen in the next session |
|  | 1. Checked the dyad’s availability and booked next visit(s).   Note: If any indication that this has happened, can code. | The OT checked when the dyad would be available for the next visit and arranged the next visit. | **Done** | OT checked when the dyad would be available for the next visit and arranged the next visit |
|  |  |  | **Done to some extent** | OT checked when the dyad would be available but did not arrange the next visit  **Or**  OT arranged the next visit but did not check the dyad were available |
|  |  |  | **Not done** | OT did not check when the dyad would be available or arrange the next visit |
|  |  |  |  |  |

| **Coding guidelines for Session 3: Ethnographic interview** | | | | |
| --- | --- | --- | --- | --- |
| **Framework component** | **Appointment activity** | **Definition** | **Scores** | **Rationale for scores** |
| **Key information** | 1. Described what will happen in the current session. | Described what will happen in the current session (Ethnographic interview, activity observation, environmental observation)   - **Ethnographic interview:** This session involves a chat with the family carer to gain information about their life story | **Done** | OT described what will happen in the current session |
|  |  |  | **Done to some extent** | OT gave a session label but does not describe what will happen in the current session |
|  |  |  | **Not done** | OT did not describe what will happen in the current session |
| **Assessment** | 1. Assessed the home environment and reported it on the checklist.   Note: Cannot tell if recording on checklists therefore code content | Assessing the home environment is when the OT looks around the home environment and assesses it. Talking/Asking about the environment is not sufficient. But, if there is evidence that the OT goes to do a home assessment this can be coded (for example: ‘I will just have a look around your home’). **Measured using OT checklist. (JB will tell us if this has been done)**  **If you have coded ‘done’ for this component in first session, choose not done (N/A)** | **Done** | OT assessed the home environment and recorded this on the COTiD-UK checklist |
|  |  |  | **Done to some extent** | OT assessed some but not all of the home environment.  **Or**  OT assessed home environment but did not record it on the checklist |
|  |  |  | **Not done** | OT did not conduct a home assessment |
| **Communication techniques** | 1. Used open ended questions.   *Note: Use ethnographic cards for scoring this | OT used open questions to ask about the family carer’s:   - Experiences - Current support and help - Experience of carrying out own activities - Experiences relating to daily schedule - Experiences relating to home environment - Dealing with the person with dementia’s behaviour/coping strategies   Note: questions must be open questions  An open question is a question that cannot have a ‘yes/no answer’. For example. ‘Did you enjoy x?’ would be a closed question, but ‘How did you find doing x?’ would be an open question. | **Done** | OT used open questions to ask about at least 5 topics |
|  |  |  | **Done to some extent** | OT used open questions to ask about 2-4 topics |
|  |  |  | **Not done** | OT used open questions to ask about none or one topic. |
|  | 1. Prompted the family carer to provide more details about their responses. | When the family carer provides an answer to a question, the OT has asked a further question to prompt the participant to go into more details about their answer. For example: ‘Can you explain a bit more?’ ‘Could you give me an example?’  **Note:** these prompts can be less subtle than this, for example if the OT asks questions to prompt more information from a certain topic. | **Done** | OT asked for more details when appropriate most of the time |
|  |  |  | **Done to some extent** | OT asked for more details **sometimes but not always**, |
|  |  |  | **Not done** | OT **did not ask for** more details |
|  | 1. Asked questions which caused distress. (*) | Questions which cause distress refer to those topics or issues that may cause undue distress to either the person, family carer or OT. Could include the continued questioning of sensitive topics, for example bereavement. | **Done** | OT asked questions which caused distress and continued questioning the person with dementia or family carer about sensitive topics. |
|  |  |  | **Not done** | OT did not ask distressing questions |
|  | 1. Used jargon or technical language. (*) | Jargon or technical language is unexplained terminology which may not be understandable to members of the general public. This includes OT terminology. For example: ‘graded’/’environmental assessment’, ‘SMART goal’, ‘OPHI’, ‘problem analysis’ ‘action planning’, ‘activity synthesis’ or condition specific terms/terminology | **Done** | OT **used** jargon or technical language 4 or more times |
|  |  |  | **Done to some extent** | OT **used** jargon or technical language 2-3 times |
|  |  |  | **Not done** | OT **did not use** jargon or technical language. |
|  | 1. Asked the family carer to explain what the meaning of situations and activities were. | OT asks the person to explain what it means to them to do a certain activity or be in a certain situation (e.g. can you tell me what doing x means to you?’).  Example: The OT asks ‘How does it make you feel when you do some gardening?’  Can be either general or specific activities | **Done** | OT asked questions on the meaning of most activities or situations |
|  |  |  | **Done to some extent** | OT asked questions on the meaning of some activities or situations |
|  |  |  | **Not done** | OT did not ask questions on the meaning of activities or situations |
|  | 1. Summarised the information provided by the family carer. | Throughout the session, the OT summarised the information provided by the family carer by putting the information into their own words  Example: ‘So you have told me that you garden two times a week and that you enjoy it’ | **Done** | OT summarised the information provided by the family carer 4 or more times |
|  |  |  | **Done to some extent** | OT summarised the information provided by the family carer 1-3 times |
|  |  |  | **Not done** | OT did not summarise the information provided by the family carer |
|  | 1. Interpreted the information provided by the family carer (*) | The OT interpreted (made an inference or assumption) the information based on what they think that the family carer is saying e.g. ‘what I think you mean is…’  Example: (After the person says they like gardening), the OT says: ‘I’m guessing that you are an outdoor person’ | **Done** | OT interpreted the information provided by the family carer more than 4 times |
|  |  |  | **Done to some extent** | OT interpreted the information provided by the family carer 3 or 4 times |
|  |  |  | **Not done** | OT did not interpret the information or interpreted the information less than two times |
|  | 1. Checked that they understood the information provided by the family carer. | The OT asks the family carer if they understood the information correctly e.g. ‘Is that right?’ ‘Did I understand that correctly?’  Example: (After the person says they like gardening), the OT says: ‘Am I right in thinking that you enjoy gardening?’ | **Done** | OT checked that they understood the information provided by the family carer 4 or more times |
|  |  |  | **Done to some extent** | OT checked they understood the information provided by the family carer 2-3 times |
|  |  |  | **Not done** | OT did not check they understood the information provided by the family carer or checked the information provided by the family carer once |
|  | 1. Spoke about themselves. (*) | The OT told the family carers about their own life. (e.g. ‘I always quite enjoyed doing x, until…’). This must be more than just a natural response to a remark, e.g. (P: I like chocolate, OT: Me too!). | **Done** | OT spoke about themselves in detail more than 4 times |
|  |  |  | **Done to some extent** | OT spoke about themselves in detail 3 to 4 times |
|  |  |  | **Not done** | OT did not speak about themselves or spoke about themselves less than two times |
|  | 1. Told the family carer what to do. (*) | The OT told the family carer what to do rather than helping them to decide what to do for themselves e.g. ‘you should do this…’ | **Done** | OT told the person what to do |
|  |  |  | **Not done** | OT did not tell the person what to do |
|  | 1. Used visual objects found in the family carer’s home to gather information (E.g. photos, garden, sewing, cakes, paintings). | Visual objects could include photos, gardens, sewing, cakes, and paintings.  The OT prompted/led the use of participants’ objects or used objects to gather information if the opportunity comes up (e.g. participant shows object to OT). | **Done** | OT prompted the use of objects in conversation to gather information |
|  |  |  | **Done to some extent** | OT initiates but does not facilitate the use of objects  Or  OT facilitates but does not initiate the use of objects |
|  |  |  | **Not done** | OT did not use objects |
| **Support** | 1. Prompted the family carer to speak. | Prompted the family carer to speak, for example: ask questions or give views.  **Note:** If family carer speaks a lot without prompting, choose ‘not done and write N/A’  Note: This is different from ‘prompting for more information’. This component refers more to whether the participant has asked the participant if they have any questions (e.g. at the end of the session), or for more information on their views on decisions  E.g. ‘Do you have any questions?’/’What’s your views on that?’  Note: If one example of stopping speaking (to move the session on), can still code ‘done’, code ‘done to some extent’ if multiple examples of stopping speaking. | **Done** | OT prompted family carer to speak on **most appropriate occasions** |
|  |  |  | **Done to some extent** | OT prompted family carer to speak on some but not all appropriate occasions |
|  |  |  | **Not done** | OT did not prompt family carer to speak |
| **Next steps** | 1. Described what will happen in the next session. | Described what will happen in the next session (Summaries and goal setting)   - **Summaries and goal setting**: This session involves the OT summarising the information gathered in the OPHI and ethnographic interviews and also their own observations from assessments and then using this information to help the dyad set goals to work on throughout the programme | **Done** | OT described what will happen in the next session |
|  |  |  | **Done to some extent** | OT gave a session label but does not describe what will happen in the next session |
|  |  |  | **Not done** | OT did not describe what will happen in the next session |
|  | 1. Checked the dyad’s availability and booked next visit(s).   Note: If any indication that this has happened, can code. | The OT checked when the dyad would be available for the next visit and arranged the next visit. | **Done** | OT checked when the dyad would be available for the next visit and arranged the next visit |
|  |  |  | **Done to some extent** | OT checked when the dyad would be available but did not arrange the next visit  **Or**  OT arranged the next visit but did not check the dyad were available |
|  |  |  | **Not done** | OT did not check when the dyad would be available or arrange the next visit |
|  |  |  |  |  |

| **Coding guidelines for Session 4/5: Summaries and goal setting** | | | | |
| --- | --- | --- | --- | --- |
| **Framework component** | **Appointment activity** | **Definition** | **Scores** | **Rationale for scores** |
| **Key information** | 1. Described what will happen in the current session. | Described what will happen in the current session   - Summaries and goal setting: This session involves the OT summarising the information gathered in the OPHI and ethnographic interviews and also their own observations from assessments and then using this information to help the dyad set goals to work on throughout the programme | **Done** | OT described what will happen in the current session |
|  |  |  | **Done to some extent** | OT gave a session label but does not describe what will happen in the current session |
|  |  |  | **Not done** | OT did not describe what will happen in the current session |
| **Feedback** | 1. Summarised the information provided in the interview with the person with dementia. | Throughout the session, the OT summarised the information from the interview with the person with dementia by putting the information into their own words  Example: ‘So you have told me that you garden two times a week and that you enjoy it’ | **Done** | OT summarised the information provided by the person with dementia 4 or more times |
|  |  |  | **Done to some extent** | OT summarised the information provided by the person with dementia 1-3 times |
|  |  |  | **Not done** | OT did not summarise the information provided by the person with dementia |
|  | 1. Checked that they understood the information provided by the person with dementia. | OT checks throughout the session if they have understood the information summarised from the interview with the person with dementia correctly e.g. ‘Is that right?’ ‘Did I understand that correctly?’  Example: (After the person says they like gardening), the OT says: ‘Am I right in thinking that you enjoy gardening?’ | **Done** | OT checked that they understood the information provided by the person with dementia 4 or more times |
|  |  |  | **Done to some extent** | OT checked they understood the information provided by the person with dementia 2-3 times |
|  |  |  | **Not done** | OT did not check they understood the information provided by the person with dementia or checked the information provided by the person with dementia once |
|  | 1. Summarised the information provided in the interview with the family carer. | Throughout the session, the OT summarised the information from the interview with the family carer by putting the information into their own words  Example: ‘So you have told me that you garden two times a week and that you enjoy it’ | **Done** | OT summarised the information provided by the family carer 4 or more times |
|  |  |  | **Done to some extent** | OT summarised the information provided by the family carer 1-3 times |
|  |  |  | **Not done** | OT did not summarise the information provided by the family carer. |
|  | 1. Checked that they understood the information provided by the family carer. | OT checks throughout the session if they have understood the information summarised from the interview with the family carer correctly e.g. ‘Is that right?’ ‘Did I understand that correctly?’  Example: (After the person says they like gardening), the OT says: ‘Am I right in thinking that you enjoy gardening?’ | **Done** | OT checked that they understood the information provided by the family carer 4 or more times |
|  |  |  | **Done to some extent** | OT checked they understood the information provided by the family carer 2-3 times |
|  |  |  | **Not done** | OT did not check they understood the information provided by the person with dementia or checked the information provided by the family carer once |
|  | 1. Summarised their own views from observations and assessments. | Summarised their own views from the observations and assessments (e.g. activity/home assessments) and **not information gathered in the interviews.** The OT should provide details about the home assessment/activity assessment and other relevant aspects e.g. challenges/achievements | **Done** | OT gave a detailed summary of their own views from the observations or assessments |
|  |  |  | **Done to some extent** | OT gave a brief summary of their own views from the observations or assessments |
|  |  |  | **Not done** | OT did not summarise their own view |
| **Activity selection** | 1. Discussed potential activities using prepared cards which listed activities and possible goals.   Note: Cannot tell if using cards, code if OT analyses potential activities | This refers to whether the OT has used activity cards to discuss potential types of activities that the dyad could engage in | **Done** | OT discussed potential activities with the dyad |
|  |  |  | **Not done** | OT did not discuss potential activities with the dyad |
| **Goal setting** | 1. Set at least one individual or joint goal for the person with dementia and family carer | Goals will specify an activity that they would like to work on. Goals can be aimed at the person with dementia, family carer or dyad.  Setting a goal includes specifying on the form who the goal is set by and what the goal is  Goals should be reported by the OT on the goal setting form. Code this component based on whether there is evidence of a goal been set on the goal setting form. | **Done** | Set at least one individual or joint goal for the person with dementia and family carer (including who the goal was set by and what the goal is) |
|  |  |  | **Done to some extent** | Set a goal but did not specify either who the goal was set by or what the goal is |
|  |  |  | **Not done** | Did not set a goal for the person with dementia and family carer |
|  | 1. Developed these goals into SMART goals | A SMART goal has the following criteria:   - Specific (what, with, who, where, and when) - Measurable (specifies how progress is measured) - Achievable (explicit outcome is mentioned) - Realistic - Timed (by when)   SMART can be discussed with family carer or created and reported on goal setting form by OT.  ***Can be supported with information from goal setting form** | **Done** | Developed at least one goal that meets **all** 5 SMART criteria and at least 3 specific criteria. |
|  |  |  | **Done to some extent** | OT and the participant developed a goal that meets **2-4** of the SMART criteria and at least 2 specific criteria |
|  |  |  | **Not done** | Did not develop a goal that included none or one of the SMART criteria. |
| **Activity adaptation** | 1. Adapted the activities to suit participants’ needs   Note: Only code if goals have been set | To adapt an activity, OTs may have identified an easily achievable activity, broken down the activity to identify the skills required, graded the activity (made it easier or more difficult), adapted the activity (equipment or materials) or designed a sequence of activities. | **Done** | OT adapted the activity/activities to suit participants needs |
|  |  |  | **Done to some extent** | OT adapted the activity/activities but it is not clear whether it is to suit participants needs |
|  |  |  | **Not done** | OT and the participant did not adapt an activity |
| **Environmental adaptation** | 1. Provided information about the environmental barriers for the dyad. | OT provides information about environmental barriers for the dyad that have been discussed in sessions/observed in assessments  Environmental barriers can be barriers in the community or home. | **Done** | OT provided information about the environmental barriers for the dyad |
|  |  |  | **Done to some extent** | OT provided information about the environmental barriers but barriers were not specific to the dyad |
|  |  |  | **Not done** | OT did not provide information about the environmental barriers |
|  | 1. Recommended changes to the dyads’ environment. | This refers to the OT making suggestions of ways that the dyad could change the physical, social, institutional environment.  **Note**: If observational assessment has been carried out and no recommendations are made, can code not applicable. (Jane to look at checklist and let us know if observational assessment has been conducted) | **Done** | OT recommended changes to the dyads’ environment |
|  |  |  | **Not done** | OT did not recommend changes to the dyads’ environment |
| **Summary** | 1. Summarised the final list of agreed goals. | The OT summarised the goals that the dyad have set in this session.  **Note:** If summarising areas of goals this can be coded. | **Done** | OT summarised all goals that have been set |
|  |  |  | **Done to some extent** | OT summarised some but not all goals |
|  |  |  | **Not done** | OT did not summarise the goals that were set. |
|  | 1. Told participants that they could start to carry out activities to meet the goals. | The OT told or encouraged participants to begin carrying out activities to meet the goals | **Done** | OT told dyad they can start to do the activities to meet the goals |
|  |  |  | **Not done** | OT did not tell the dyad that they can start to work on the goals. |
| **Support** | 1. Prompted the person with dementia and family carer to speak. | Prompted the person with dementia and family carer to speak, for example: ask questions or give views.  **Note:** If person with dementia and family carer speak a lot without prompting, choose ‘not done and write N/A’  Note: This is different from ‘prompting for more information’. This component refers more to whether the participant has asked the participant if they have any questions (e.g. at the end of the session), or for more information on their views on decisions  E.g. ‘Do you have any questions?’/’What’s your views on that?’  Note: If one example of stopping speaking (to move the session on), can still code ‘done’, code ‘done to some extent’ if multiple examples of stopping speaking. | **Done** | OT prompted person with dementia and family carer to speak on **most appropriate occasions** |
|  |  |  | **Done to some extent** | OT prompted person with dementia and family carer to speak on some but not all appropriate occasions |
|  |  |  | **Not done** | OT did not prompt person with dementia and family carer to speak |
| **Next steps** | 1. Described what will happen in the next session. | Described what will happen in the next session (Consultation and advice), or whatever skill session the OT are providing next.   - **Consultation and advice:** This session involves a chat with the family carer to work out what is working well and what is not working and to support the family carer to think about how to achieve those goals. | **Done** | OT described what will happen in the next session |
|  |  |  | **Done to some extent** | OT gave a session label but does not describe what will happen in the next session |
|  |  |  | **Not done** | OT did not describe what will happen in the next session |
|  | 1. Checked the dyad’s availability and booked next visit(s).   Note: If any indication that this has happened, can code. | The OT checked when the dyad would be available for the next visit and arranged the next visit. | **Done** | OT checked when the dyad would be available for the next visit and arranged the next visit |
|  |  |  | **Done to some extent** | OT checked when the dyad would be available but did not arrange the next visit  **Or**  OT arranged the next visit but did not check the dyad were available |
|  |  |  | **Not done** | OT did not check when the dyad would be available or arrange the next visit |
|  |  |  |  |  |

| **Coding guidelines for Session 6: Consultation and advice** | | | | |
| --- | --- | --- | --- | --- |
| **Framework component** | **Appointment activity** | **Definition** | **Scores** | **Rationale for scores** |
| **Key information** | 1. Described what will happen in the current session. | Described what will happen in the current session (Consultation and advice)   - **Consultation and advice:** This session involves a chat with the family carer to work out what is working well and what is not working and to support the family carer to think about how to achieve those goals. | **Done** | OT described what will happen in the current session |
|  |  |  | **Done to some extent** | OT gave a session label but does not describe what will happen in the current session |
|  |  |  | **Not done** | OT did not describe what will happen in the current session |
|  | 1. Explained that they can offer support to the dyad but cannot solve their problems for them. | Explained that they are able to offer support (or help the dyad) but cannot solve the dyad’s problems for them.  Note: If the OT makes it clear in the session that they are supporting/helping the dyad and not telling them what to do, can code | **Done** | OT explained that they can support the partnership between the dyad but cannot solve problems |
|  |  |  | **Done to some extent** | OT explained that they can support but did not explain they cannot solve problems  **Or**  OT explained they cannot solve problems but did not explain that they can support the partnership, |
|  |  |  | **Not done** | OT did not explain they can support but cannot solve problems |
| **Operationalising goals** | 1. Prompted family carer to select an action to help them achieve their goals | Prompted the family carer to select an action to help them achieve their goals. This could include thinking about what they can do to achieve goals and choosing an action to work on | **Done** | OT prompted the family carer to think about what they can do to achieve their goals and choose an action |
|  |  |  | **Done to some extent** | OT prompted the family carer to think about what they can do to achieve the goals but not to choose an action  Or  OT prompted the family carer to choose an action but did not discuss whether this would help them to achieve their goals |
|  |  |  | **Not done** | OT did not prompt the family carer to think about what they can do to achieve the goals or choose an action |
| **Problem analysis** | 1. Prompted the family carer to identify things that have helped the dyad to do their activity/activities. | Prompted the family carer to talk about things that have helped them to do the activity.  Note: As long as things that helped are discussed (either initiated by OT or family carer), can be coded. | **Done** | OT prompted the family carer to identify things that helped them to do the activity/activities |
|  |  |  | **Not done** | OT did not prompt the family carer to identify things that helped them to do the activity/activities |
|  | 1. Prompted the family carer to identify barriers that prevented the dyad from doing their activity/activities | Prompted the family carer to talk about barriers that prevented the dyad from doing their activity/activities  Note: As long as barriers are discussed (either initiated by OT or family carer), can be coded. | **Done** | OT prompted the family carer to identify barriers that prevented the dyad from doing the activity/activities |
|  |  |  | **Not done** | OT did not prompt the family carer to identify barriers that prevented the dyad from doing the activity/activities |
|  | 1. Prompted the family carer to identify solutions for these problems. | Prompted the family carer to identify specific solutions for goals that haven’t been achieved, or general solutions to general problems.  Note: As long as solutions are discussed (either initiated by OT or family carer), can be coded. | **Done** | Prompted the family carer to identify solutions |
|  |  |  | **Not done** | Did not prompt the family carer to identify solutions |
| **Key information** | 1. Provided information about dementia. | Provided the carer with information about dementia (can include behavioural features of dementia).  When discussing behavioural features OT may provide and explain Alzheimer’s fact sheet on topics including sleep, agitation, hallucinations in relation to the person’s situation | **Done** | OT provided all information about dementia when needed/appropriate |
|  |  |  | **Done to some extent** | OT provided some but not all information about dementia when needed/appropriate |
|  |  |  | **Not done** | OT did not provide information about dementia when needed/appropriate |
|  | 1. Provided information about support available to the dyad (e.g. community resources). | Support refers to specific support to the dyad and issues that they are facing, in relation to dementia. For example: signposting to community resources. | **Done** | OT provided information about support available to the dyad |
|  |  |  | **Done to some extent** | OT provided some information about support  **Or**  OT provided information but did not make the information specific to the dyad |
|  |  |  | **Not done** | OT did not provide information about support |
| **Communication techniques** | 1. Prompted the person to provide more details about their responses. | When participants have provided an answer to a question, the OT has asked a further question to prompt the participant to go into more details about their answer. For example: ‘Can you explain a bit more?’ ‘Could you give me an example?’ Note these prompts can be less subtle than this, for example if the OT asks questions to prompt more information from a certain topic. | **Done** | OT asked for more details when appropriate most of the time |
|  |  |  | **Done to some extent** | OT asked for more details **sometimes but not always**, |
|  |  |  | **Not done** | OT **did not ask for** more details |
|  | 1. Used jargon or technical language. (*) | Jargon or technical language refers to unexplained language which may not be understandable to members of the general public. This includes OT terminology. For example: ‘graded’/’environmental assessment’, ‘SMART goal’, ‘OPHI’, ‘problem analysis’ ‘action planning’, ‘activity synthesis’ or condition specific terms/terminology | **Done** | OT **used** jargon or technical language 4 or more times |
|  |  |  | **Done to some extent** | OT **used** jargon or technical language 2-3 times |
|  |  |  | **Not done** | OT **did not use** jargon or technical language. |
|  | 1. Prompted the family carer to express their feelings. | Prompted the family carer to tell the OT how they are feeling and what their reactions to situations are. | **Done** | OT sufficiently prompted the family carer to express their feelings and feelings were expressed |
|  |  |  | **Done to some extent** | OT prompted the family carer to express their feelings but did not follow through appropriately |
|  |  |  | **Not done** | OT did not prompt the family carer to express their feelings |
| **Summarise** | 1. Provided a summary of the activities discussed. | The OT summarised the activities that were discussed in this session (session summary) | **Done** | OT summarised all activities that were discussed |
|  |  |  | **Done to some extent** | OT summarised some but not all activities that were discussed |
|  |  |  | **Not done** | OT did not summarise the activities that were discussed |
| **Support** | 1. Prompted the family carer to speak. | Prompted the family carer to speak, for example: ask questions or give views.  **Note:** If family carer speaks a lot without prompting, choose ‘not done and write N/A’  Note: This is different from ‘prompting for more information’. This component refers more to whether the participant has asked the participant if they have any questions (e.g. at the end of the session), or for more information on their views on decisions  E.g. ‘Do you have any questions?’/’What’s your views on that?’  Note: If one example of stopping speaking (to move the session on), can still code ‘done’, code ‘done to some extent’ if multiple examples of stopping speaking. | **Done** | OT prompted family carer to speak on **most appropriate occasions** |
|  |  |  | **Done to some extent** | OT prompted family carer to speak on some but not all appropriate occasions |
|  |  |  | **Not done** | OT did not prompt and family carer to speak |
| **Next steps** | 1. Described what will happen in the next session. | Described what will happen in the next session (Evaluation)   - **Evaluation:** This session consists of reviewing the behavioural goals and helping the dyad to identify ways to continue making progress after the programme. | **Done** | OT described what will happen in the next session |
|  |  |  | **Done to some extent** | OT gave a session label but does not describe what will happen in the next session |
|  |  |  | **Not done** | OT did not describe what will happen in the next session |
|  | 1. Checked the dyad’s availability and booked next visit(s).   Note: If any indication that this has happened, can code. | The OT checked when the dyad would be available for the next visit and arranged the next visit. | **Done** | OT checked when the dyad would be available for the next visit and arranged the next visit |
|  |  |  | **Done to some extent** | OT checked when the dyad would be available but did not arrange the next visit  **Or**  OT arranged the next visit but did not check the dyad were available |
|  |  |  | **Not done** | OT did not check when the dyad would be available or arrange the next visit |

| **Coding guidelines for Session 7: Evaluation** | | | | |
| --- | --- | --- | --- | --- |
| **Framework component** | **Appointment activity** | **Definition** | **Scores** | **Rationale for scores** |
| **Key information** | 1. Described what will happen in the current session. | Described what will happen in the current session (evaluation)   - Evaluation: This session consists of reviewing the behavioural goals and helping the dyad to identify ways to continue making progress after the programme. | **Done** | OT described what will happen in the current session |
|  |  |  | **Done to some extent** | OT gave a session label but does not describe what will happen in the current session |
|  |  |  | **Not done** | OT did not describe what will happen in the current session |
| **Review goals** | 1. Reviewed the behavioural goal(s) using the COTiD-UK goal setting form. | Review behaviour goals involves asking about progress towards meeting the goals (doesn’t require evidence of using the form as recording it on the form is the next apt activity) | **Done** | OT reviewed the behavioural goals with the participant |
|  |  |  | **Done to some extent** | OT partly reviewed the behavioural goals |
|  |  |  | **Not done** | OT did not review the behavioural goals |
|  | 1. Recorded the review of the goal using the COTiD-UK goal setting form. | The OT has recorded the review of the goal on the COTiD-UK goal setting form.  **Note**: Use the goal setting form to answer this | **Done** | OT recorded the review on the COTiD-UK goal setting form |
|  |  |  | **Done to some extent** | OT partly recorded the review on the COTiD-UK goal setting form |
|  |  |  | **Not done** | OT did not record the review on the COTiD-UK goal setting form |
| **Planning ahead** | 1. Prompted the dyad to identify long term goals for how to continue making progress. | The OT prompted the dyad to think about and choose long term goals for making progress after the intervention  **Note:** Can be areas of goals as well as specific goals.  This component focuses more on discussing **what** the dyad can work on after the programme | **Done** | OT helped the dyad to identify goals to help them to continue making progress and goals were agreed. |
|  |  |  | **Not done** | OT did not help the dyad to identify goals to continue making progress |
|  | 1. Suggested ways in which the dyad can continue making progress. | The OT has given suggestions of ideas on how the dyad can begin to work on these goals.  This component focuses more on discussing **how** the dyad can work on their goals – e.g. making suggestions. | **Done** | OT suggested ways for how the dyad can continue to make progress |
|  |  |  | **Done to some extent** | OT partly suggested ways but did not develop these fully |
|  |  |  | **Not done** | OT did not suggest ways they can continue making progress |
| **Support** | 1. Prompted the person with dementia and family carer to speak. | Prompted the person with dementia and family carer to speak, for example ask questions or give views.  **Note:** If person with dementia and family carer speak a lot without prompting, choose ‘not done and write N/A’  Note: This is different from ‘prompting for more information’. This component refers more to whether the participant has asked the participant if they have any questions (e.g. at the end of the session), or for more information on their views on decisions  Note: If one example of stopping speaking (to move the session on), can still code ‘done’, code ‘done to some extent’ if multiple examples of stopping speaking. | **Done** | OT prompted person with dementia and family carer to speak on **most appropriate occasions** |
|  |  |  | **Done to some extent** | OT prompted person with dementia and family carer to speak on some but not all appropriate occasions |
|  |  |  | **Not done** | OT did not prompt person with dementia and family carer to speak |
| **Next steps** | 1. Explained what will happen next. | The OT explained what would happen next (as they had just finished the last session). E.g. the researchers will come for another visit. | **Done** | OT explained what would happen next |
|  |  |  | **Done to some extent** | OT partly explained what would happen next |
|  |  |  | **Not done** | OT did not explain what would happen next |
|  | 1. Told the dyad not to tell the researcher that they received the COTiD-UK intervention. | The OT told the dyad not to tell the researcher who comes to the 12 week follow up that they received the COTiD-UK intervention (to keep the blinding) | **Done** | OT told the dyad not to tell the researcher that they received the intervention |
|  |  |  | **Not done** | OT did not tell the dyad not to tell the researcher that they received the intervention |
