# Supplementary material for: Measuring fidelity of delivery of the Community Occupational Therapy in Dementia-UK intervention
Source: BMC Geriatr. 2019 Dec 23;19:364. doi: 10.1186/s12877-019-1385-7 (PMC6929510; doi:10.1186/s12877-019-1385-7)

**Additional file 3 – COTiD-UK checklists**

Introduction checklist


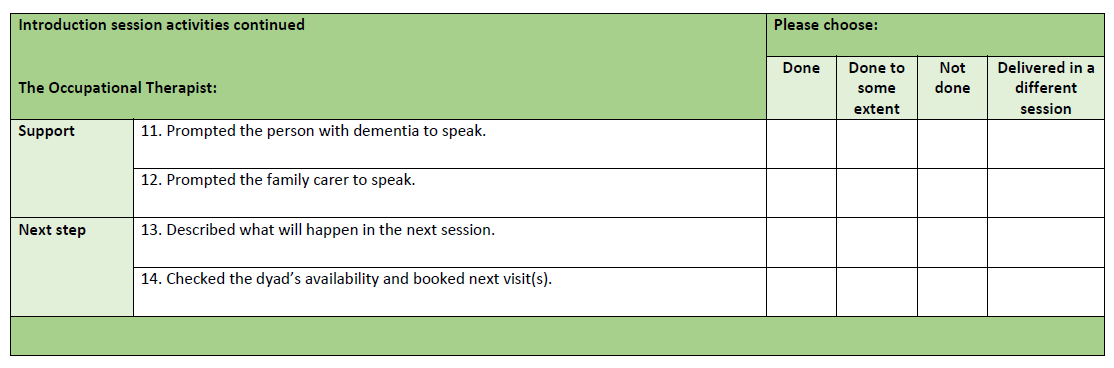

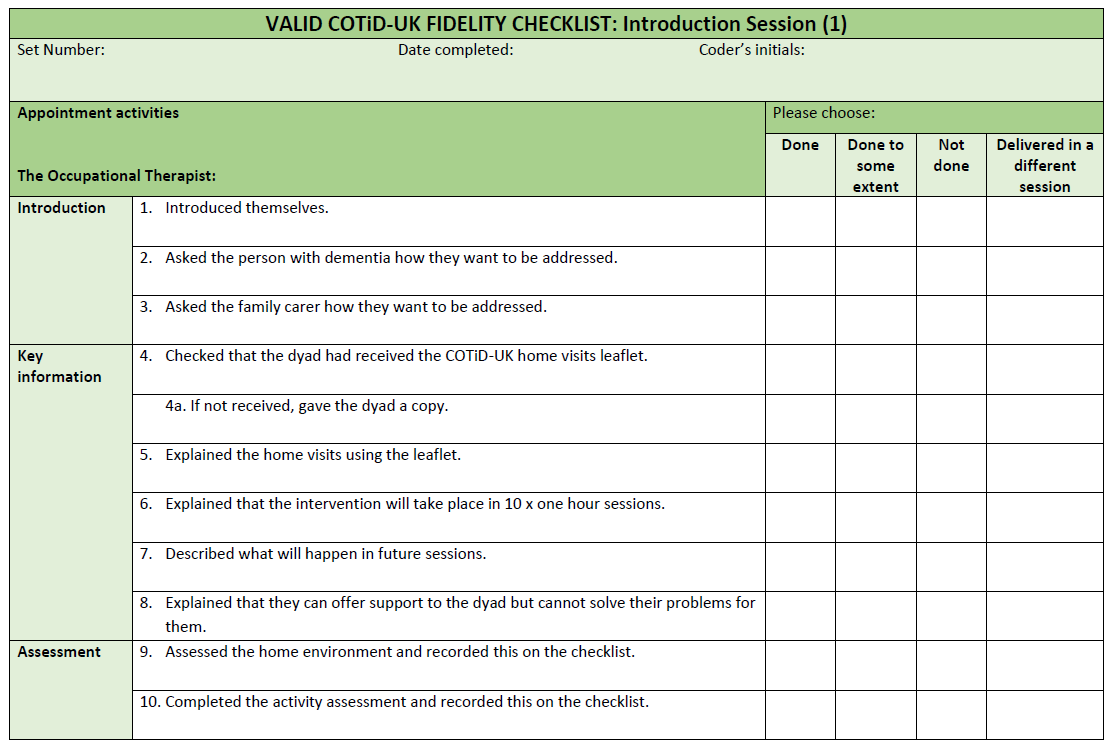


OPHI checklist


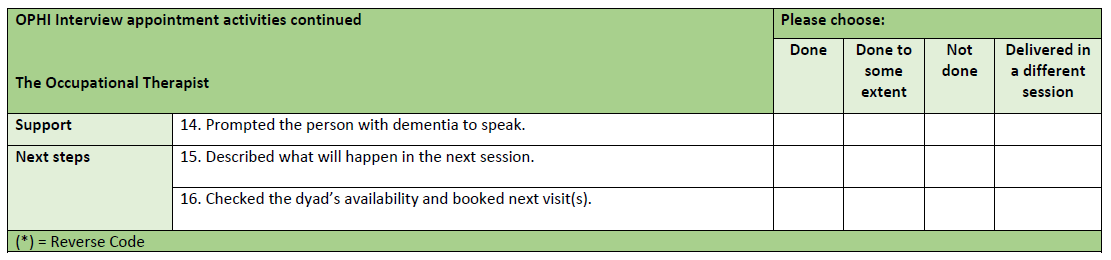

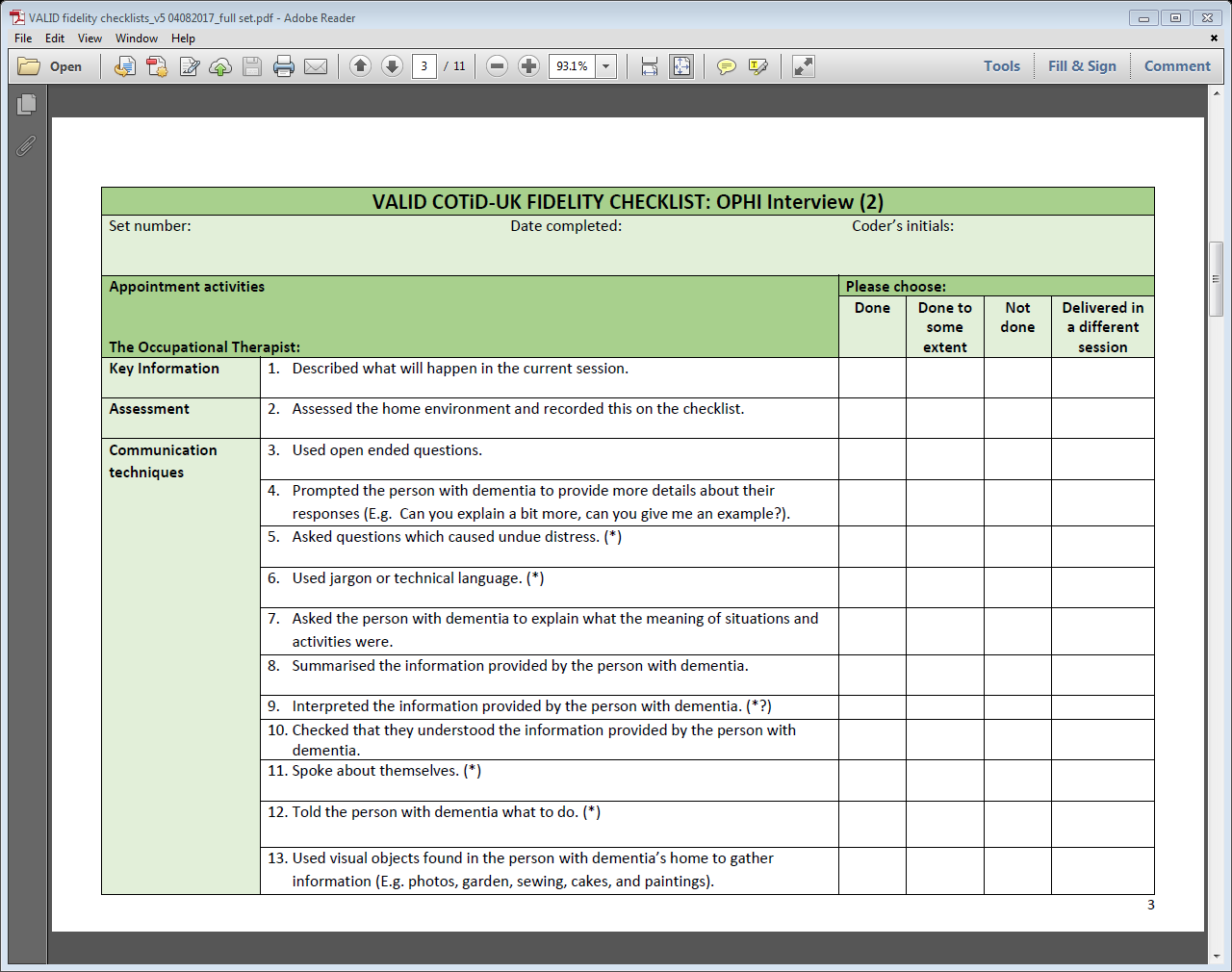


Ethnographic interview checklist


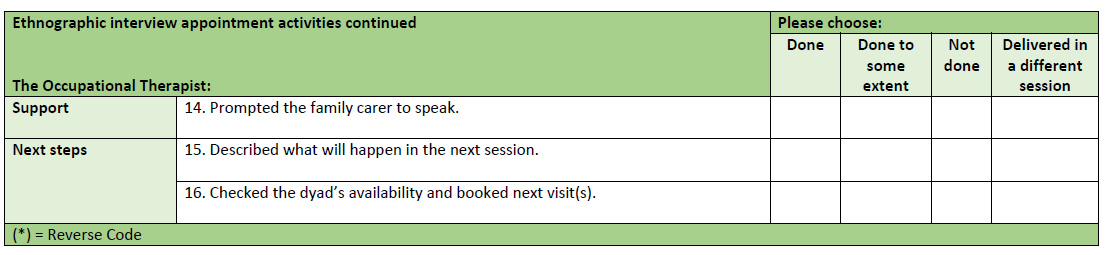

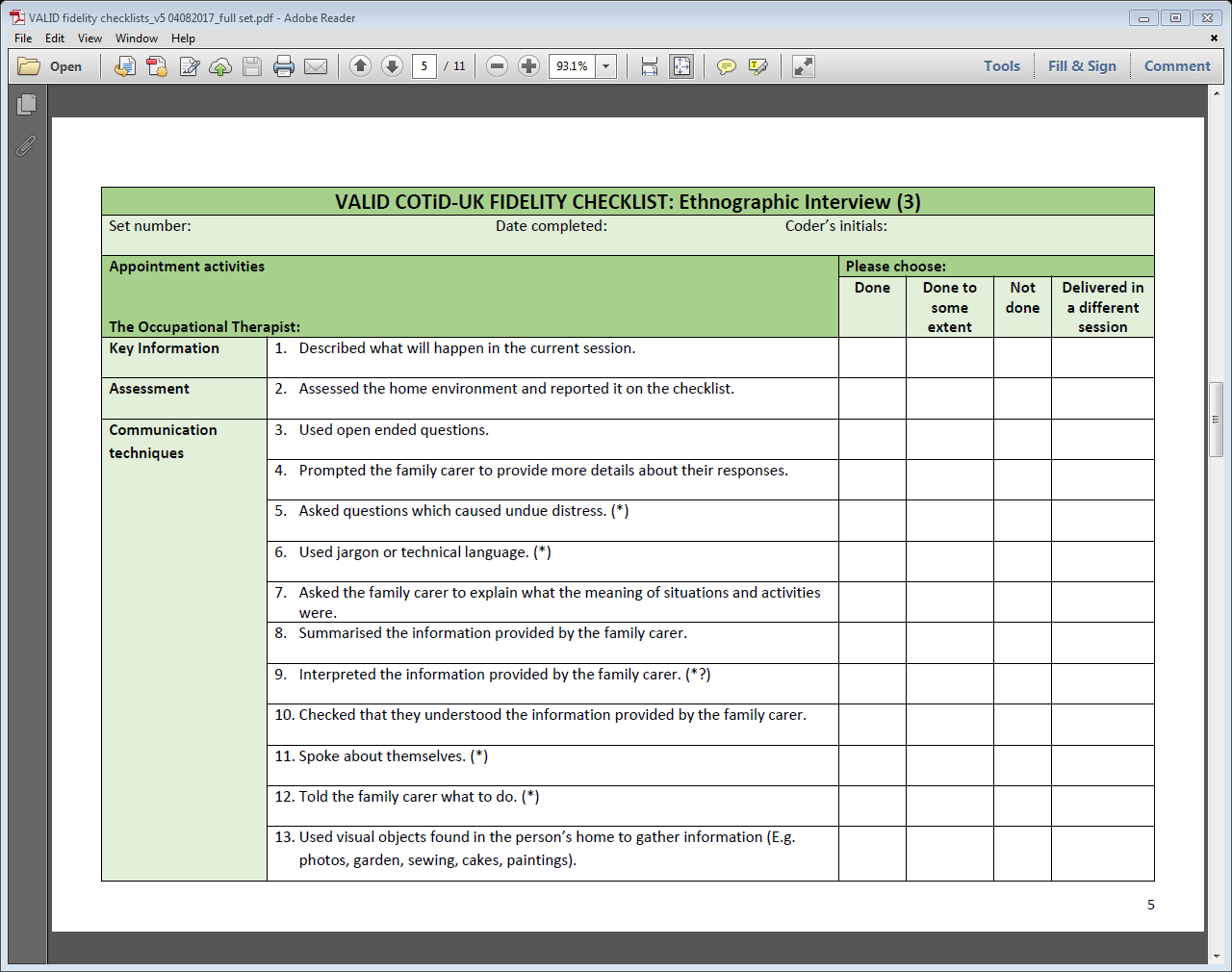


Goal setting and summary checklist


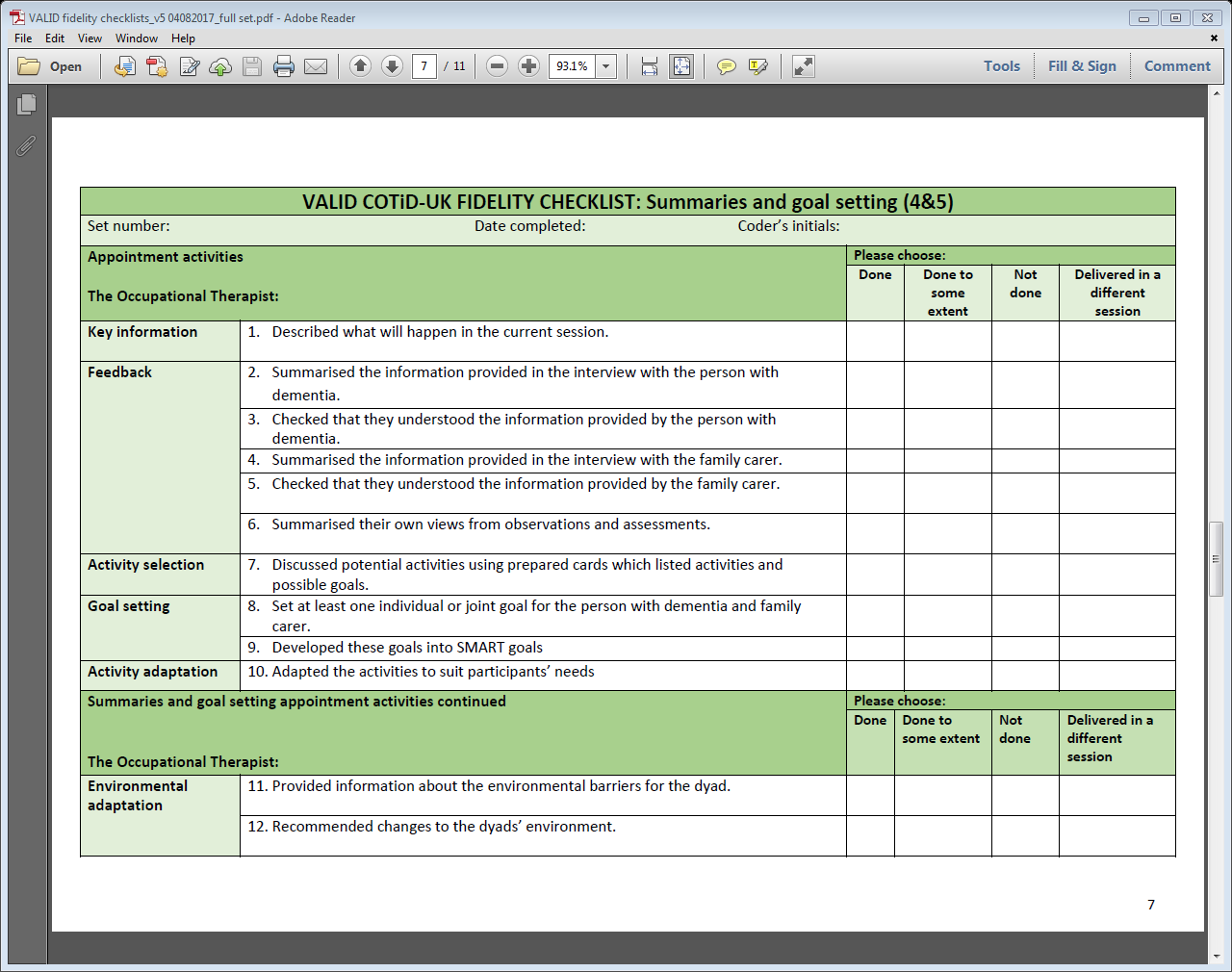

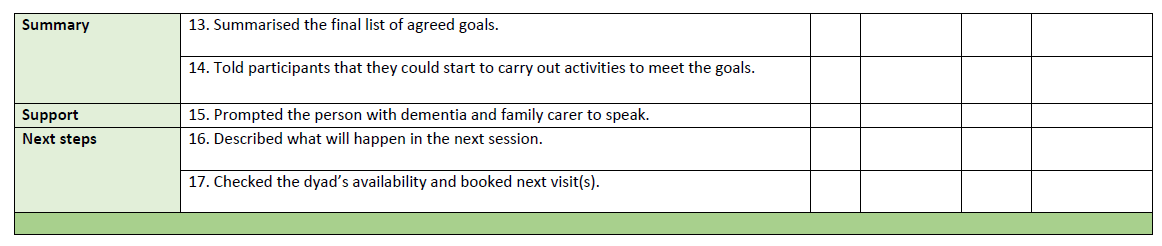


Consultation and advice checklist


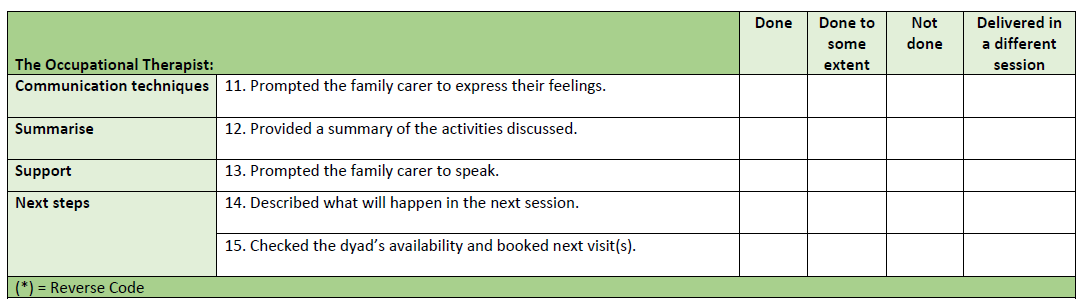

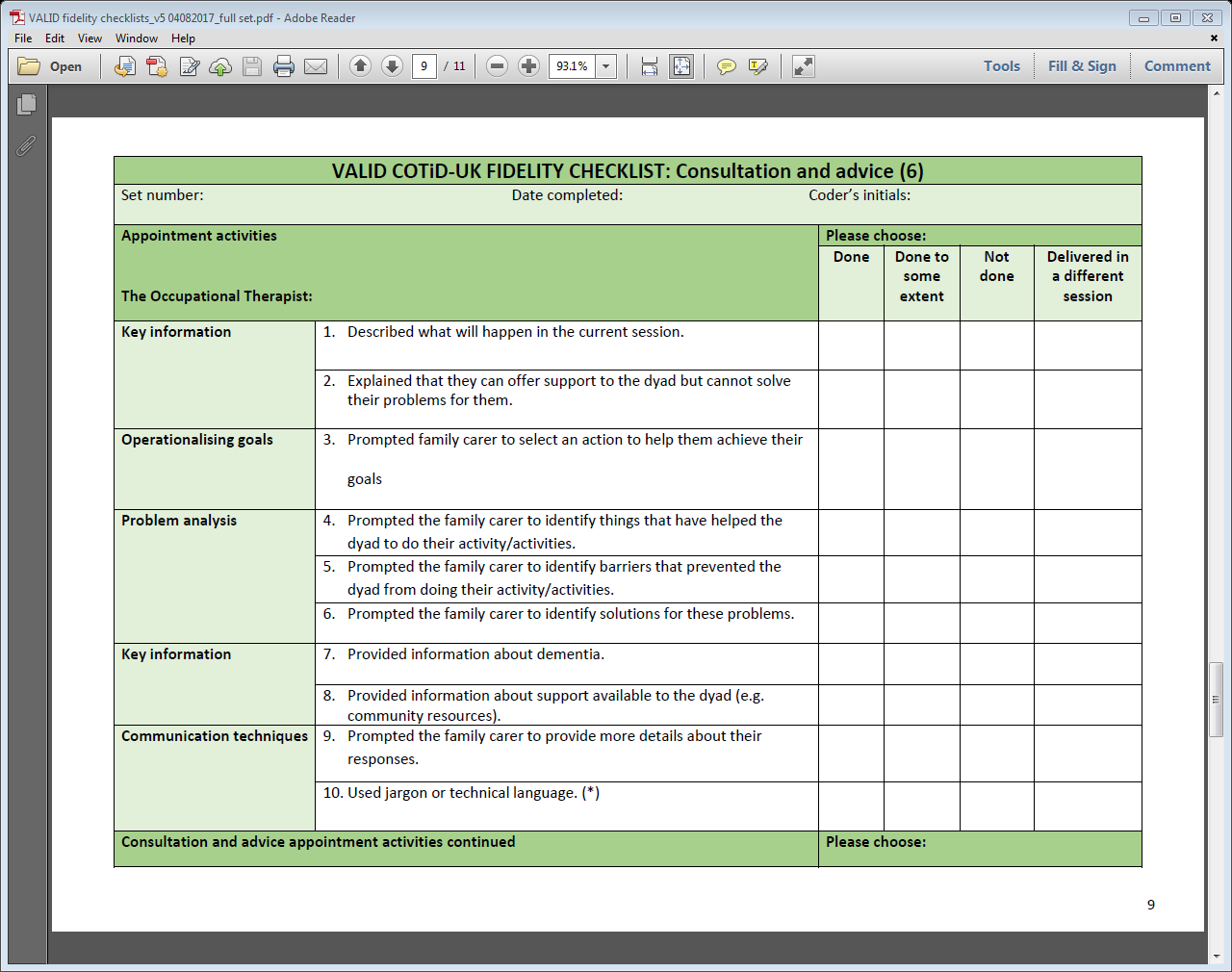


Evaluation checklist


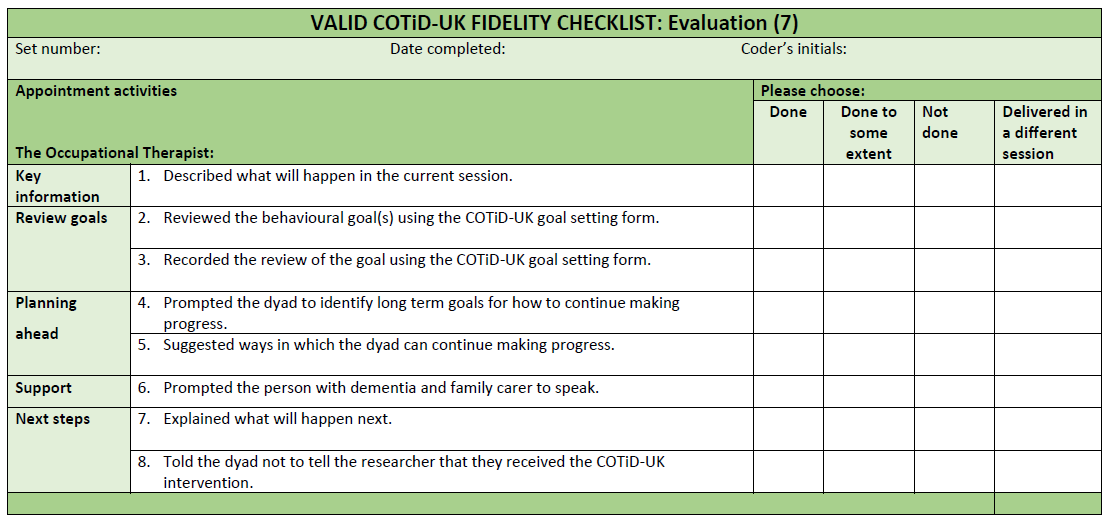

Supplement: Supplementary file 3 — Additional file 3. COTiD-UK checklists [file 12877_2019_1385_MOESM3_ESM.docx]
