# Supplementary material for: Measuring fidelity of delivery of the Community Occupational Therapy in Dementia-UK intervention
Source: BMC Geriatr. 2019 Dec 23;19:364. doi: 10.1186/s12877-019-1385-7 (PMC6929510; doi:10.1186/s12877-019-1385-7)

Additional file 4. Percentage of transcripts in which individual components were delivered fully, to some extent, or not at all per COTiD-UK sessions


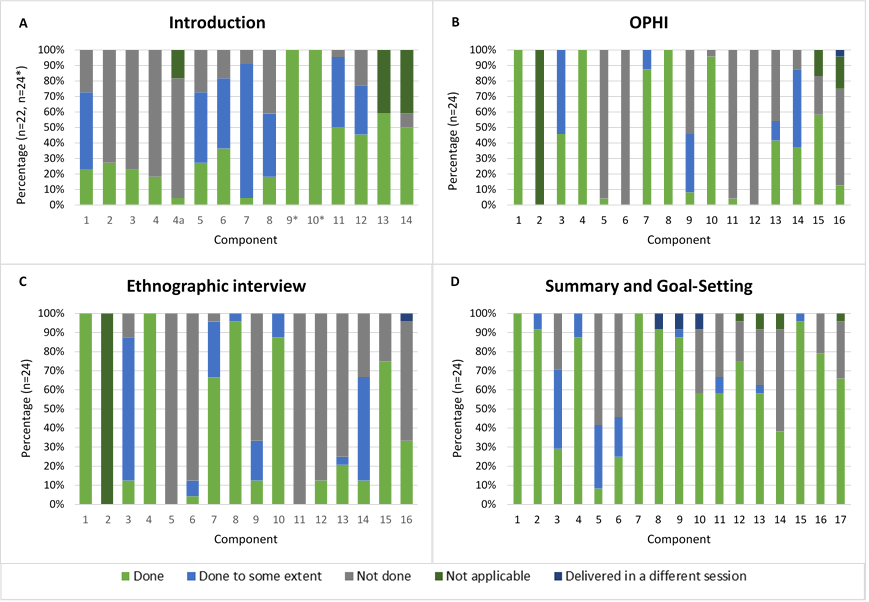


Additional file 4. Continued


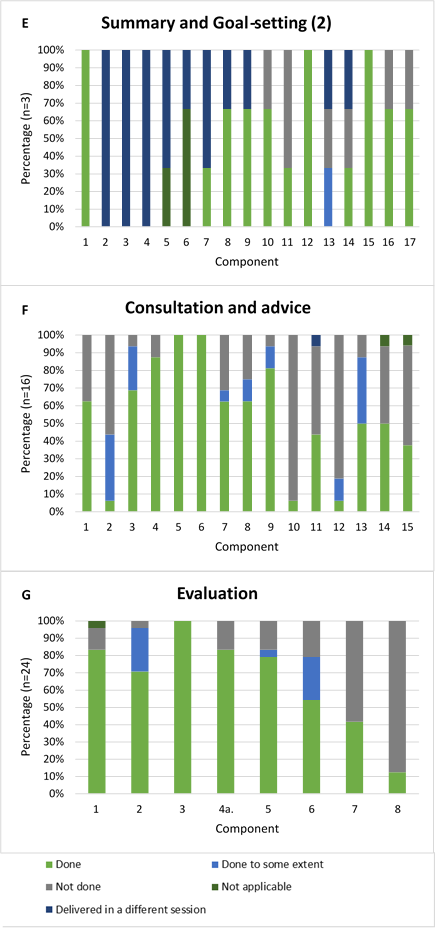

Supplement: Supplementary file 4 — Additional file 4. Percentage of transcripts in which individual components were delivered fully, to some extent, or not at all per COTiD-UK sessions [file 12877_2019_1385_MOESM4_ESM.docx]
